# Black Sea-Derived Biomaterials for Wound-Healing Applications

**DOI:** 10.3390/ijms27115066

**Published:** 2026-06-03

**Authors:** Emin Cadar, Florentina Nicoleta Roncea, Adrian Cosmin Roșca, Ana-Maria Peșterău, Cristina-Crenguța Albu, Lucia Bubulac, Laura Ana-Maria Drăgan, Sanda Jurja, Claudia Florina Bogdan-Andreescu, Iuliana Stoicescu, Rodica Sirbu

**Affiliations:** 1 Faculty of Pharmacy, “Ovidius” University of Constanta, Capitan Aviator Al. Serbanescu Street, No. 6, Campus, Corp C, 900470 Constanta, Romaniaiulianapse@yahoo.com (I.S.); 2 Organizing Institution for Doctoral University Studies of “Carol Davila”, Faculty of Pharmacy, “Carol Davila” University of Medicine and Pharmacy, Dionisie Lupu Street, No. 37, Sector 2, 020021 Bucharest, Romania; 3 Department of Genetics, Faculty of Dentistry, “Carol Davila” University of Medicine and Pharmacy, 020021 Bucharest, Romania; 4 Department of Family Medicine, Faculty of Medicine, “Carol Davila” University of Medicine and Pharmacy, 020021 Bucharest, Romania; lucia.bubulac@umfcd.ro; 5 Faculty of Medicine, “Ovidius” University of Constanta, University Alley, No. 1, Campus, Building B, 900470 Constanta, Romania; 6 Faculty of Dental Medicine, Department of Speciality Disciplines, “Titu Maiorescu” University, 031593 Bucharest, Romania; claudia.andreescu@prof.utm.ro

**Keywords:** wound repair, molecular pathways, regenerative biology, tissue remodeling, Black Sea bioresources, biopolymer composites

## Abstract

Wound healing is a complex, multi-stage process governed by tightly regulated molecular mechanisms. However, effective regenerative therapies remain with limitations. This study presents a novel marine-derived biocomposite, JPC-ALG-CT, designed to improve wound healing through synergistic bioactive mechanisms. The material incorporates collagen extracted from the jellyfish *Rhizostoma pulmo*, chitosan derived from the crab *Pachygrapsus mormoratus*, and polysaccharide-rich extracts from the green alga *Cladophora vagabunda*, all sourced from the Black Sea. The study is based on the biochemical analysis of these three marine-derived components, highlighting the collagen content of jellyfish, the polysaccharides present in algae, and the bioactive properties of chitosan. The biochemical and physico-chemical properties of each component were characterized, with particular emphasis on the structural features of jellyfish collagen and the functional bioactivity of chitosan and algal polysaccharides. The research findings are supported by the identification of the collagen type extracted from jellyfish, as well as by the characterization of chitosan and green algal extracts. The resulting composite demonstrated significant antioxidant and antimicrobial activities, indicating its potential to integrate key processes involved in wound repair, including inflammation control and microbial protection. In vitro studies using fibroblast and keratinocyte models showed that the JPC-CT-ALG biocomposite supported cell viability at lower tested concentrations and promoted scratch closure in cell monolayers, suggesting preliminary wound-relevant biological activity. These findings suggest that the combined marine-derived components interact to enchance wound healing at the cellular level. This work evidenced the potential of marine biomaterials as sources for next-generation regenerative therapies and supports further investigation into their molecular mechanisms and in vivo applications for improved wound care.

## 1. Introduction

Wound healing is a complex process of major concern to both patients and the medical field, wound progression being associated with increased morbidity and in severe cases lead to mortality [1,2]. Wounds represent anatomical disruptions of the skin that can also involve deeper tissues and structures, including blood vessels, subcutaneous tissue, tendons, muscles, nerves, and, in severe cases, bone [3].

### 1.1. Acute Wounds and Chronic Wounds

Wounds are commonly divided into acute and chronic types. Acute wounds usually heal within about four weeks and follow a relatively predictable sequence of events: hemostasis, inflammation, proliferation, and remodeling [4,5]. While this sequence is well described in acute wound repair, chronic wounds remain much more difficult to manage. In these cases, there is often a clear discrepancy between the knowledge from experimental and clinical research about the mechanisms of healing and the results achieved with standard treatment approaches. Chronic wounds do not progress through the normal healing stages in an orderly manner, and their evolution is influenced by a wide range of local and systemic factors [6].

For this reason, a improved understanding of the molecular mechanisms involved in wound repair is essential for developing better therapies. In acute wounds, the biological events associated with each phase of healing are generally well understood and can be followed during the repair process [7]. However, when this process is disturbed, healing may become abnormal. This can result either in excessive scar formation, such as hypertrophic scars and keloids, or in wounds that fail to heal properly, including ulcers and other chronic lesions [8]. In chronic wounds, healing is substantially delayed, the normal healing phases are not properly coordinated, and the process can be complicated by wound infection [9].

Conventional wound-healing treatments focus on systemic optimization and local wound management [10,11]. Also, new therapeutic interventions have emerged, including skin substitutes produced through tissue engineering, hyperbaric oxygen therapy, negative-pressure wound therapy, and cellular or acellular matrix-like products [12,13,14,15]. A comprehensive understanding of wound-healing physiology is therefore necessary in order to improve the management of wounds and scars and to minimize complications associated with scarring [16,17].

### 1.2. Materials from Marine Resources Used in Wound Healing

Conventional treatments used for acute and chronic wound healing mostly rely on antibiotics and analgesics, which mainly help to reduce infection and relieve pain rather than actively stimulate tissue regeneration [18,19]. However, chronic wounds remain associated with a high risk of morbidity, which has encouraged the search for new therapeutic strategies based on bioactive natural compounds with wound-healing potential [20]. As a result, increasing attention has been given to alternative treatments that use natural compounds to support and accelerate the repair process [21]. In addition to medicinal plants, marine-derived natural products have also gained interest as potentially resources for wound-healing applications [22]. Among these compounds, collagen has been widely investigated for skin tissue repair and has been incorporated into different formulations, including gels, pastes, powders, and wound dressings [23,24,25]. In previous years, marine collagen has been proposed as an alternative to collagen obtained from terrestrial sources, such as porcine and bovine tissues, due to concerns related to disease transmission, including transmissible spongiform encephalopathies, potential viral contamination, and foot-and-mouth disease [26]. The interest in collagen-based materials for tissue regeneration is also supported by their reported antioxidant and antimicrobial properties [27,28,29]. Consequently, several studies have explored the use of collagen in the development of products intended for skin disorders and wound-healing applications [30,31,32]. Marine collagen extracted from jellyfish has attracted a lot of interest in recent years, mainly because several studies have described its properties and potential relevance for biomedical applications [33,34]. In the case of the jellyfish *Rhizostoma pulmo*, antioxidant activity has also been reported by different research groups [35,36,37]. Previous studies have explored jellyfish collagen-based hydrogels combined with extracts from the brown alga *Cystoseira barbata* for wound-healing applications, as well as collagen–seaweed extract formulations proposed for the treatment of skin and oral mucosal disorders [38].

However, collagen extracts obtained from jellyfish generally contain only small quantities of polysaccharides [39]. For this reason, seaweed-derived extracts may be useful in wound-healing formulations, as they can enrich the material with polysaccharides known to participate in several stages of the repair process [40]. Chitosan, another marine-derived polysaccharide obtained from the chitin of marine crabs, has also received considerable attention in this field [41]. Several authors have reported chitosan-based pharmaceutical formulations for wound-healing applications [42,43,44]. The chitosan potential role in tissue regeneration has been widely investigated [45,46,47,48,49]. To increase the polysaccharide content of wound-healing formulations, green algae may represent a valuable resource due to their content of sulfated polysaccharides, compounds known to be involved in different stages of tissue repair [50,51,52]. Their relevance is further supported by several studies reporting the antioxidant and antimicrobial activities of green algae [53,54,55]. In this context, the main objective of the present study is to develop and evaluate new composite biomaterials for wound-healing applications using natural marine resources from the Black Sea. These raw materials provide important bioactive compounds, including marine collagen, chitosan, and green seaweed-derived polysaccharides. The source of the materials was the Black Sea: the collagen was extracted from the jellyfish *Rhizostoma pulmo*, the chitosan and chitin from which it was obtained come from the crab *Pachygrapsus mormoratus*, and the algal extract is from the green alga *Cladophora vagabunda*. After extraction, each marine-derived material was analyzed to determine its biochemical composition, including collagenous compounds, polyphenols, flavonoids, polysaccharides, and amino acids. These analyzes were used to support the observed antioxidant and antimicrobial activities of the individual extracts. The newly developed biocomposite, obtained by combining the three marine-derived ingredients, was evaluated for antioxidant and antimicrobial activity, as well as for in vitro biocompatibility and scratch closure using BALB/3T3 clone A31 fibroblasts and HaCaT human keratinocytes. Because the present study is limited to in vitro models, the results should be interpreted as preliminary evidence of wound-relevant biological activity. The study also intended to discuss the biological mechanisms involved in the different stages of wound healing in order to emphasize the relevance of the natural bioactive components present in the newly developed marine-derived biocomposite and their potential contribution to tissue repair.

## 2. Results

The new pharmaceutical formulations developed for wound healing and skin tissue regeneration were developed using marine resources from the Black Sea. These included 70% hydroalcoholic ethanol extracts obtained from the green alga *Cladophora vagabunda* (*C. vagabunda*), collagen peptide extracts isolated from the jellyfish *Rhizostoma pulmo* (*R. pulmo*), and chitosan extracts prepared from chitin obtained from the sea crab *Pachygrapsus mormoratus*. The marine extracts obtained from each species were first analyzed individually. Subsequently, the newly developed biocomposite, produced by combining these components, was also evaluated.

### 2.1. Biochemical Ingredients

**Figure 1:**
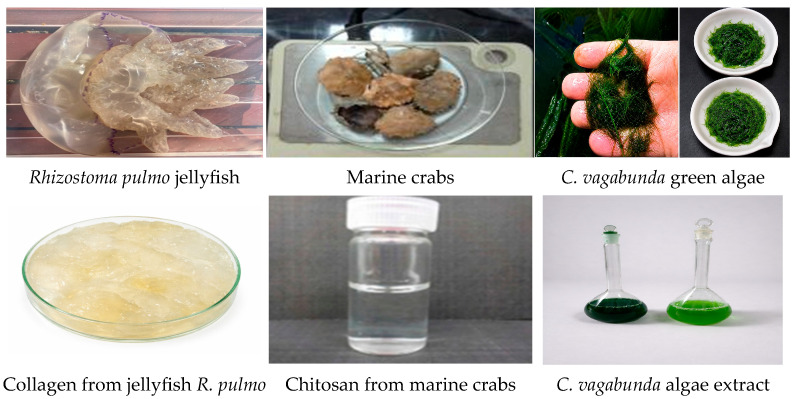
Materials from the Black Sea and extracts obtained from these materials.

The ingredients used in this study were obtained from marine resources and are presented in Figure 1. Jellyfish collagen was extracted from the Black Sea jellyfish *R. pulmo* using the PSC method, resulting in both hydrolyzed collagen and jellyfish collagen peptides. Hydroalcoholic extracts were prepared from the green alga *C. vagabunda*, while chitosan hydrogel was obtained from the stone crab *Pachygrapsus mormoratus*.

### 2.2. Physico-Chemical Characteristics of the Ingredients

#### 2.2.1. Marine Collagen from Jellyfish

**Figure 2:**
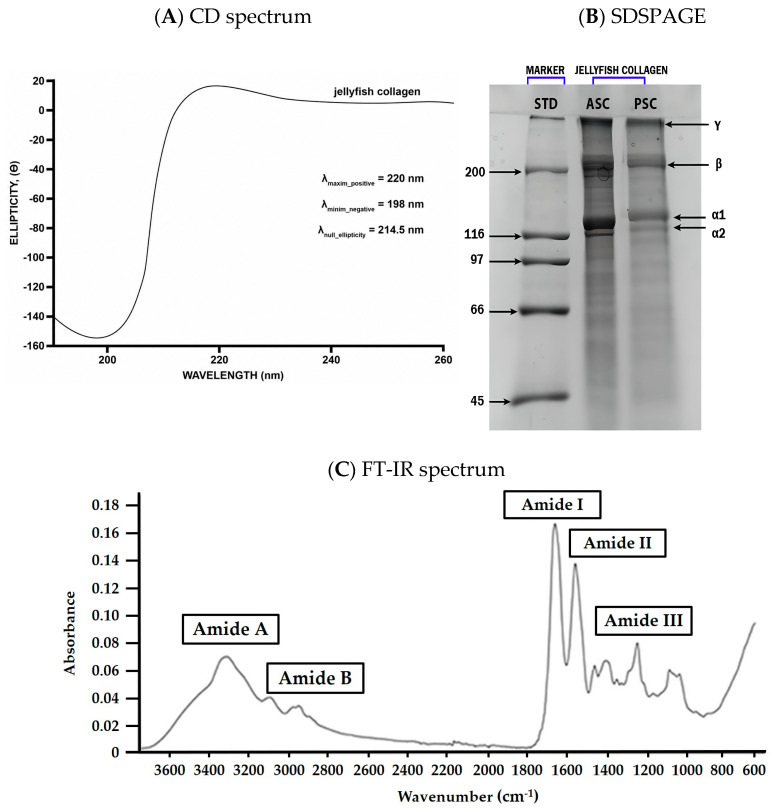
Physico-chemical characterization of *R. pulmo* collagen: (**A**) CD spectrum; (**B**) SD−AGE analysis; and (**C**) FTIR spectrum of jellyfish collagen.

Collagen extracted from the jellyfish *Rhizostoma pulmo* (*R. pulmo*) was characterized through several physico-chemical analyses, including circular dichroism (CD), SDS-PAGE, and FT-IR spectroscopy. The results of these analyses are shown in Figure 2 and confirm the structural characteristics of the jellyfish-derived collagen.

Although these analyses differ in procedure (circular dichroism spectrum, SDS-PAGE analysis, and FT-IR spectrum), they were all performed on collagen extracted from the *R. pulmo* jellyfish in the Black Sea, and the results were then used to characterize the physico-chemical properties of this collagen. Jellyfish collagen was extracted using a validated method and was used to produce biocomposites with other ingredients, as previously published [38,56]. Marine collagen extracted from marine fish has also been studied by many researchers [30,32,57]. The structural data obtained indicated a type I triple-helix collagen structure [38,56,57].

#### 2.2.2. Structural Data for Marine Chitosan from Black Sea Stone Crabs

**Figure 3:**
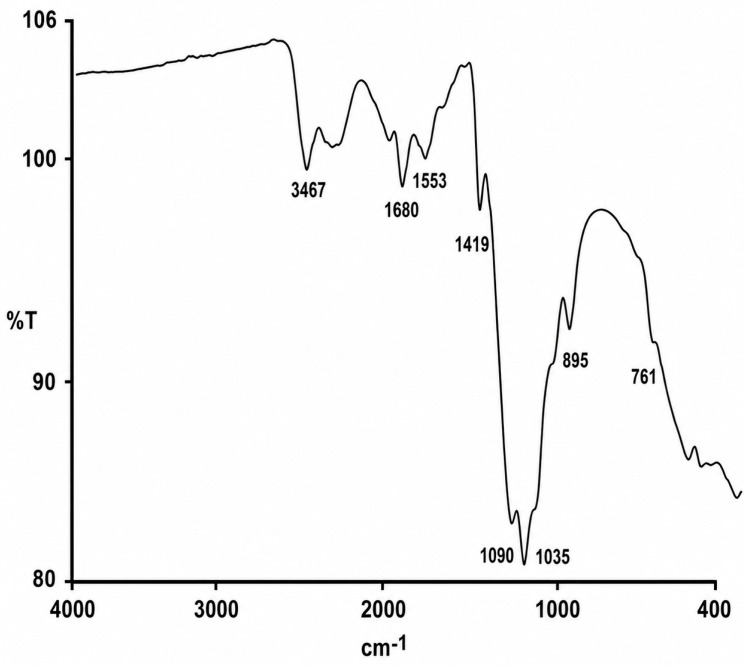
FT-IR spectrum for the chitosan sample.

Chitin and its derivative, chitosan, are important marine-derived polysaccharides. Their molecular structures, together with the FT-IR analysis of chitosan, are presented in Figure 3.

### 2.3. Composite Data for Marine Ingredients from the Black Sea

#### 2.3.1. Biocomposition Data for Green Algae ALG and Collagen Peptides (JPCs)

The proximate composition of the green alga *C. vagabunda*, jellyfish collagen from *R. pulmo*, and marine chitosan is presented in Table 1. Reference values are also included for comparison, both for the composition of the algal extracts and for the jellyfish collagen peptide composition obtained from *R. pulmo*.

The results obtained for *C. vagabunda* are compared with those reported by Cadar et al. in 2025 and Sirbu et al. in 2020 [53,54]. The composition of jellyfish collagen is compared with the values reported by Pesterau et al. in 2025 [38]. Marine algae are characterized by a high content of carbohydrates, including sulfated compounds, whereas jellyfish collagen is distinguished by its high protein content.

#### 2.3.2. Physico-Chemical Characteristics for Marine Chitosan

Marine chitosan was obtained from the stone crab *Pachygrapsus mormoratus* through the conversion of chitin into chitosan. The extracted chitosan was characterized using several specific physico-chemical parameters, including moisture content, ash content, degree of deacetylation (DD), molecular weight, pH, and solubility. The results are presented in Table 2, together with reference data from the literature [47].

#### 2.3.3. Amino-Acid Content

The results for the amino acid content of jellyfish collagen are presented in Table 3, where results reported by James et al. and Pesterău et al. for the amino acids in collagen extracted from the jellyfish *R. Pulmo* are also included [37,38].

#### 2.3.4. Polyphenolic Content

##### TPC (Total Phenolic Content) and TFC (Total Flavonoid Content)

Marine algae also contain bioactive compounds of scientific interest, including polyphenols and flavonoids. Table 4 shows the results for TPC and TFC obtained for the green alga *C. vagabunda*, expressed per 100 g of powdered sample for TPC (mg GAE/100 g d.w.) and for TFC (mg CE/100 g d.w.). These values are compared with those reported by Cadar et al. in 2023 [50]. The results represent the mean ± SD for *n* = 3. Here, d.w. refers to dry weight.

The results can also be discussed in relation to the work of Leone et al. who investigated *R. pulmo* from the Mediterranean Sea [34]. Phenolic compounds are known to contribute to the antioxidant activity of marine-derived natural bio-compounds. The bioactive compounds obtained from the green alga *C. vagabunda* showed higher total phenolic and flavonoid contents than the collagen peptides. Similar data on the total content of phenolic and flavonoid compounds in marine extracts have also been reported in the literature [50,54,55].

##### Individual Phenolic Content

The individual polyphenols identified by HPLC analysis in both the green alga *C. vagabunda* and jellyfish collagen are presented in Table 5. For *C. vagabunda*, the results are also compared with data reported by Sirbu et al. in 2019 [55].

### 2.4. Characteristics of New Formulations F1 and F2 Intended for Wound Healing

The new biocomposite intended for wound-healing applications was prepared in two gel formulations, F1 and F2. Both formulations contained chitosan and collagen hydrogels mixed in equal proportions, 1:1 (*v*/*v*), to which a hydroalcoholic extract of the green algae *C. vagabunda* was added. The algal extract was obtained by macerating *C. vagabunda* in 70% ethyl alcohol for 10 days. It was then incorporated into the biocomposite at two different concentrations, 10% and 20%. Based on these concentrations, two formulations, F1 and F2, were prepared, as described in Table 6.

#### 2.4.1. Microscopic Study

**Figure 4:**
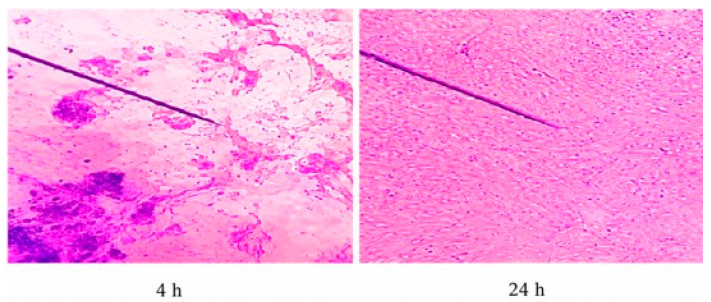
Photomicrographs at 200 µm with the evolution of composition homogeneity for the new formulation made from marine ingredients.

The new biocomposite designated for wound-healing applications was prepared in two gel formulations, F1 and F2. Both formulations contained chitosan and collagen hydrogels mixed in equal proportions, 1:1 (*v*/*v*), to which a hydroalcoholic extract of the green algae *C. vagabunda* was added. The algal extract was obtained by macerating *C. vagabunda* in 70% ethyl alcohol for 10 days. It was then incorporated into the biocomposite at two different concentrations, 10% and 20%. Based on these concentrations, two formulations, F1 and F2, were prepared, as described in Table 6 and the microscopic study at 200 µm is presented in Figure 4.

Microscopic images showed a gradual color change as the interactions between the components of formulations F1 (JPC-CT-ALG 10%) and F2 (JPC-CT-ALG 20%) became stabilized. Both formulations had a viscous, gel-like consistency. After 4 h, the components were only partially homogenized, and the images still showed color differences associated with the individual constituents of the mixture. After 24 h, the more uniform color distribution indicated that the formulations had reached a higher degree of homogenization.

#### 2.4.2. Rheological Study

**Figure 5:**
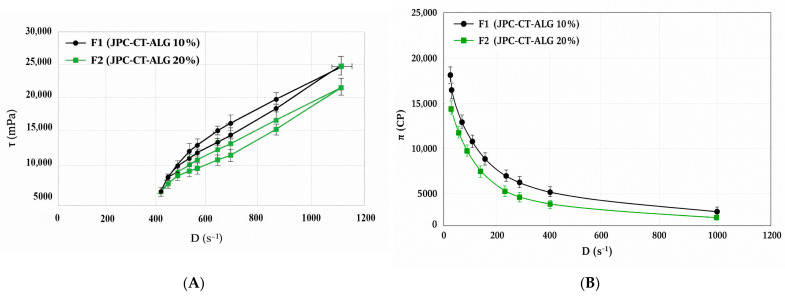
Rheological evaluation based on the rheological parameters established for formulations F1 (JPC-CT-ALG 10%) and F2 (JPC-CT-ALG 20%) of the new biocomposite upon increasing and decreasing the shear-rate gradient. (**A**) Rheogram for F1 and F2 biocomposites. (**B**) Flow curve for F1 and F2 biocomposites.

To evaluate the stability of the new wound-healing formulations, rheological studies were performed by analyzing the rheograms and flow curves, together with the corresponding rheological parameters. Figure 5 presents the rheograms and flow curves obtained for the two biocomposite formulations, F1 (JPC-CT-ALG 10%) and F2 (JPC-CT-ALG 20%). The rheological analysis demonstrated that the new gel formulations based on marine-derived bio-compounds exhibited pseudoplastic behavior across the entire range of shear rates tested. The rheograms and flow curves showed a relatively uniform profile, with a hysteresis loop observed between the increasing and decreasing shear-rate phases. Similar rheological behavior has been reported by other authors for collagen-based hydrogels [25,38,58]. Overall, the prepared hydrogels showed rheological characteristics consistent with the Ostwald–de Waele model.

### 2.5. Antioxidant Activity

#### 2.5.1. DPPH Test

**Figure 6:**
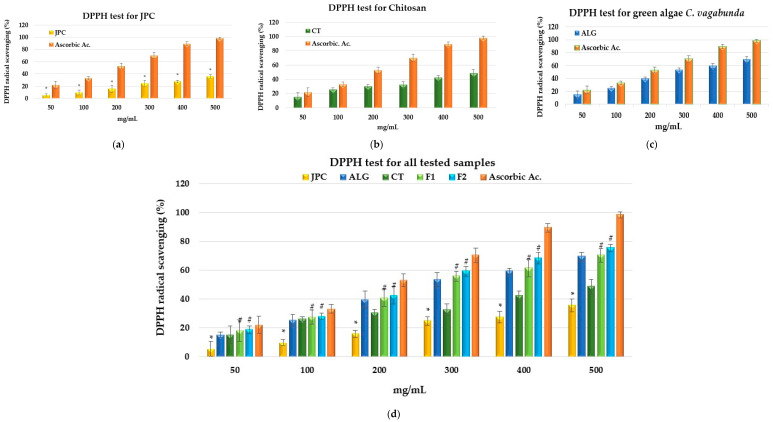
DPPH test for all ingredients, F1 and F2 bio-composites. Statistical significance was noted with * *p* < 0.05 for JPC scompared to ascorbic acid standard; # *p* < 0.05 for F1 and F2 compared to JPC. (**a**) DPPH assay for JPC; (**b**) DPPH assay for chitosan (CT); (**c**) DPPH assay for green algae (ALG); (**d**) DPPH for JPCs, ALG, CT, and bio-composites F1 and F2.

The antioxidant activity of the samples was evaluated using the DPPH assay. The results obtained for antioxidant activity using this method are shown in Figure 6a–d. Figure 6a shows the results for collagen peptides of JPC. Figure 6b and Figure 6c present the antioxidant activity of the chitosan extract (CT) and the hydroalcoholic extract obtained from the green alga *C. vagabunda* (ALG), respectively. Figure 6d compares the antioxidant activity of the individual components, JPC, CT, and ALG, with that of the two biocomposite formulations, F1 (JPC-CT-ALG 10%) and F2 (JPC-CT-ALG 20%). One-way ANOVA followed by a *t*-test was used to identify statistically significant differences between F1, F2, and the individual components. The results were marked with * for the comparison between JPC and ascorbic acid and with # for the comparison between the biocomposites F1 and F2 and JPC, with significance set at *p* < 0.05.

The results showed that the new biocomposites, F1 and F2, exhibited antioxidant activity close to that of the ascorbic acid standard, suggesting an improvement compared with the individual marine-derived components. This effect may be related to the combined contribution of the bioactive compounds present in the formulations. Specifically, the antioxidant capacity can be associated with the polysaccharide content provided by chitosan and *C. vagabunda* extracts. In addition, polyphenolic compounds, flavonoids, and amino acids derived from the algal extract and jellyfish collagen peptides may also contribute to the increased antioxidant activity of the biocomposites. The IC_50_ values determined in the DPPH assay further support these observations. The IC_50_ value was 673.17 mg/mL for JPC collagen peptides, 514.4 mg/mL for chitosan extract, and 280.37 mg/mL for the hydroalcoholic extract of *C. vagabunda*. For the biocomposites, the IC_50_ values were lower, reaching 268.33 mg/mL for F1 (JPC-CT-ALG 10%) and 252.9 mg/mL for F2 (JPC-CT-ALG 20%). The ascorbic acid standard showed an IC_50_ value of 189.03 mg/mL. The lower IC_50_ values obtained for F1 and F2 compared with the individual components indicate an improved antioxidant effect, particularly in the formulation containing 20% algal extract.

#### 2.5.2. Testul Reducing Power

**Figure 7:**
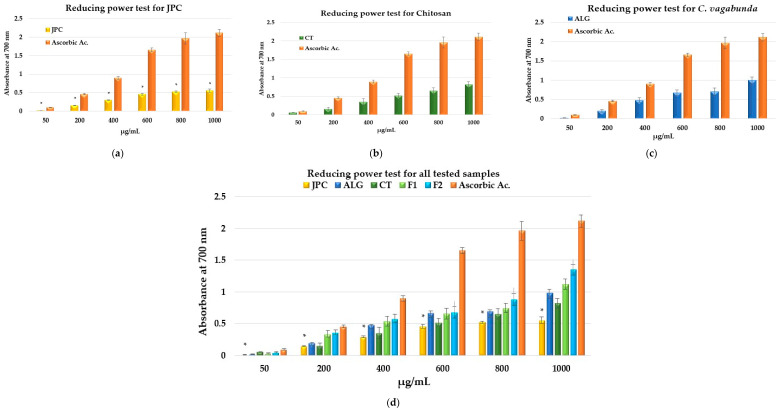
Reducing power for all biocomposites F1 and F2 and for ingredients > JPC, CT and ALG. Statistical significance was noted with * *p* < 0.05 for JPC compared to ascorbic acid standard. (**a**) Reducing power assay for JPCs; (**b**) Reducing power assay for chitosan (CT). (**c**) Reducing power assay for green algae (ALG). (**d**) Reducing power assay for JPCs, ALG, CT, and bio-composites F1 and F2.

The antioxidant activity was also evaluated using the reducing power assay. The results obtained for the individual marine-derived ingredients are presented in Figure 7a–c: Figure 7a shows the results for jellyfish collagen peptides (JPCs), Figure 7b presents the results for chitosan (CT), and Figure 7c shows the results for the algal extract (ALG). Figure 7d compares the reducing power of the individual components, JPCs, CT, and ALG, with that of the two biocomposite formulations, F1 (JPC*CT-ALG 10%) and F2 (JPC-CT-ALG 20%). Ascorbic acid was used as the reference standard.

Statistical analysis was performed using one-way ANOVA followed by a *t*-test, and the results were considered statistically significant at *p* < 0.05. Statistical significance was marked with * for the comparison between JPCs and the ascorbic acid standard.

The data indicate that the antioxidant activity increased in the newly prepared formulations F1 and F2, obtained by combining bioactive compounds from *R. pulmo*, marine chitosan, and the green alga *C. vagabunda*.

The reducing power assay reflects the ability of antioxidant compounds to donate electrons and reduce oxidized intermediates formed during lipid peroxidation. Through this mechanism, antioxidants may act as both primary and secondary antioxidant agents [38,59,60]. In the present study, the reducing power demonstrated a concentration-dependent trend, increasing with higher sample concentrations. Figure 7d shows that the reducing power of the new preparation is increased compared to both JPC from *R. pulmo* and CT extract but also compared to algal extracts of *C. vagabunda* ALG. The reducing power was concentration-dependent and increased with increasing concentration.

The statistical analysis of the results from the Reducing Power Assay shows that the data for the F1 and F2 samples were not statistically significant in the one-way ANOVA and *t*-test at *p* < 0.05. Furthermore, the statistical analysis performed on the individual ingredients *C. vagabunda* algae and chitosan compared to the ascorbic acid standard did not yield results of statistical significance. The only ingredient that showed statistical significance was the JPC from *R. Pulmo*. The antioxidant activity of green algae supported by polyphenol compositions has been reported in other studies [50,51,52,53,54,55].

### 2.6. Amtimicrobian Activity

The antimicrobial activity was evaluated against several microbial strains, including Gram-positive bacteria, *S. aureus* (ATCC 25923) and *S. epidermidis* (ATCC 12228); Gram-negative bacteria, *K. pneumoniae* (ATCC 13883), *P. mirabilis* (ATCC 25933), and *E. coli* (ATCC 25922); and the fungal strain *C. albicans* (ATCC 10231).

**Figure 8:**
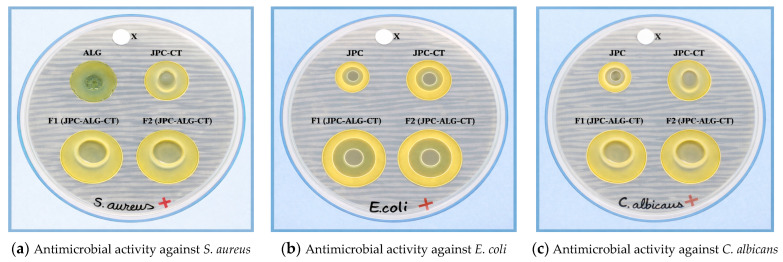
Antimicrobial activity of biocompozites F1, F2, JPC-CT mixture and JPC or ALG. (**a**) Antimicrobial activity of biocomposites F1, F2, JPC-CT mixture and *C. vagabunda* ALG algae extract against *S. aureus* X—negative control. (**b**) Antimicrobial activity of biocomposites F1, F2, JPC-CT mixture and JPC collagen peptides against *E. coli* bacteria. X—negative control. (**c**) Antimicrobial activity of biocomposites F1, F2 of the JPC-CT mixture and JPC collagen peptides against *C. albicans*. X—Negative control.

Figure 8a presents the antimicrobial activity of the F1 and F2 biocomposites, obtained from the JPC-CT mixture and the green alga *C. vagabunda* extract, against *S. aureus*.

Figure 8b presents the antimicrobial activity of the two biocomposite formulations, F1 and F2, as well as that of the JPC collagen peptides, against *E. coli*. Figure 8c presents the corresponding results obtained against *C. albicans*. In both cases, the activity of the newly developed biocomposites was compared with that of the individual components.

Overall, F1 and F2 showed clear antimicrobial effects, both in comparison with the negative control (untreated microorganisms) and relative to the separately tested ingredients. Figure 8 summarizes the antimicrobial activity expressed as the diameter of the inhibition zones (mm), determined by the diffusimetric method. This analysis was carried out for both biocomposite formulations, F1 and F2, as well as for the individual components JPC, CT, and ALG, while chloramphenicol was used as the reference standard.

The results indicate that the two formulations of the new biocomposite exhibited antimicrobial activity comparable to that of the standard. The data also suggest that the algal extract makes an important contribution to the overall antimicrobial effect of the formulations. An important antimicrobial activity was observed against *S. aureus* and *E. coli*.

**Figure 9:**
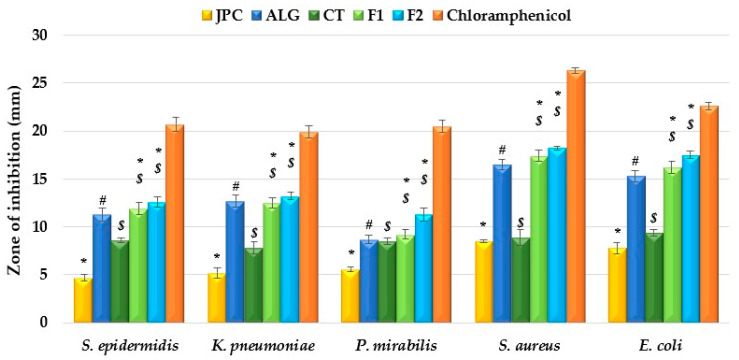
Antimicrobial activity of the biocomposite formulations F1 and F2 and their individual components, JPC, CT, and ALG. Chloramphenicol was used as the control. Results are expressed as the mean ± SD for *n* = 3. Statistical significance was considered at *p* < 0.05 and marked as follows: * for JPC compared with chloramphenicol, F1, and F2; # for ALG compared with chloramphenicol; and $ for CT compared with chloramphenicol, F1, and F2.

The antimicrobial activity results of biocomposite formulations F1 and F2 and their individual components, JPC, CT and ALG is presented in Figure 9.

In the study of antioxidant activity, the minimal inhibitory concentration (MIC) in µg/mL was also established, which we present in Table 7 and which shows values from 25 µg/mL to >100 µg/mL.

The antimicrobial activity due to marine algae, which have a large contribution, as shown in our study, has also been analysed by other researchers [61,62,63,64]. It has been demonstrated that the structure and permeability of the cell wall is the reason for the different sensitivity of Gram-positive, Gram-negative bacteria and fungi.

### 2.7. Biological Evaluations of New Composites for Wound-Healing Use

#### 2.7.1. Biological Evaluations on BALB/3T3 Fibroblasts

In vitro biological evaluations were carried out on cell lines using the scratch assay on BALB/3T3 clone A31 fibroblasts, in accordance with the ISO 10993-1 standard. This cell line was selected because fibroblasts play an essential role in the wound-healing process [65,66]. Skin tissue is mainly composed of three cell types: keratinocytes, melanocytes, and fibroblasts. Based on previous studies involving wound repair, transplantation, and cell cultures, fibroblasts and HaCaT cells are considered adequate in vitro models for epidermal studies, particularly because of their high proliferative capacity and relevance in tissue engineering [67].

**Figure 10:**
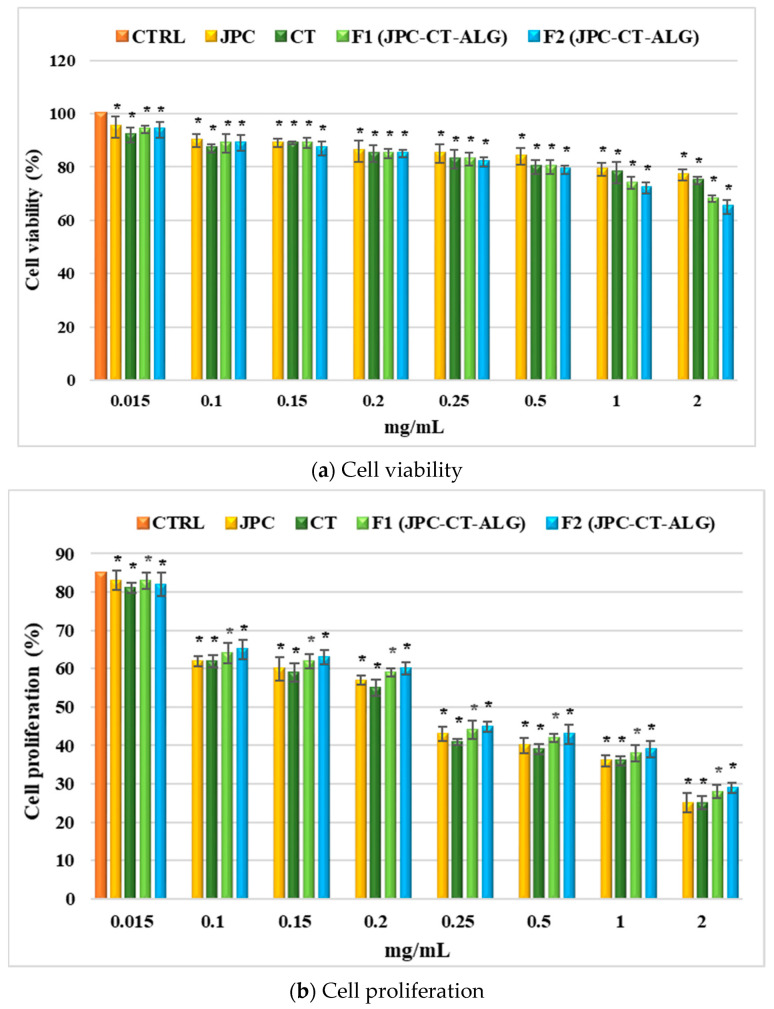
Shows the cytotoxicity tests performed on BALB/3T3 cells (clone A31) treated with JPC, CT, and the two biocomposites, F1 (JPC-CT-ALG 10%) and F2 (JPC-CT-ALG 20%), at concentrations ranging from 0.015 mg/mL to 2 mg/mL; (**a**) results after 24 h of exposure for cell viability; (**b**) results after 48 h of exposure for cell proliferation. Statistical significance was marked with * for *p* < 0.05 for all tested samples compared with the control. Untreated cells were used as the control. Each result represents the mean ± SD of eight replicates.

The biological evaluation also included cytotoxicity testing of the individual components and the final formulations. Thus, JPC collagen peptides extracted from *R. pulmo*, chitosan obtained from sea crabs, and the biocomposites F1 (JPC-CT-ALG 10%) and F2 (JPC-CT-ALG 20%), were tested. The results of the cytotoxicity evaluation performed on BALB/3T3 clone A31 fibroblasts are presented in Figure 10.

Figure 10a shows that cell viability decreased as the concentration increased for all analyzed samples. After 24 h of incubation, the results indicated good cell viability at lower concentrations; within the range of 0.015–0.2 mg/mL, viability remained above 85%. As the concentration increased, cell viability gradually declined, although values remained above 65% after 24 h for all tested samples. After 48 h of incubation, cell proliferation decreased more markedly with increasing concentration, as shown in Figure 10b. Statistical analysis using one-way ANOVA followed by the *t*-test confirmed that the results were statistically significant, as indicated by * (*p* < 0.05) for all samples compared with the control. At the lowest tested concentration, 0.015 mg/mL, all samples demonstrated cell proliferation values above 80%. At 0.5 mg/mL, proliferation decreased to 40–43%, and at 2 mg/mL it further declined to 25–29%. Similar cytotoxicity profiles have been reported by other authors for collagen peptides and seaweed-derived compounds investigated for wound-healing applications [38,40,68,69,70,71].

**Figure 11:**
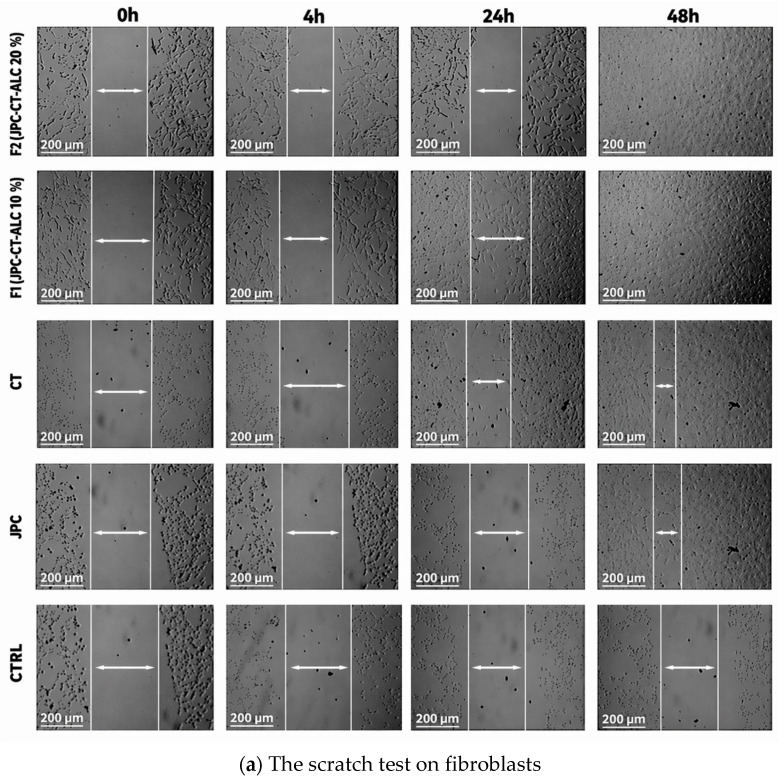

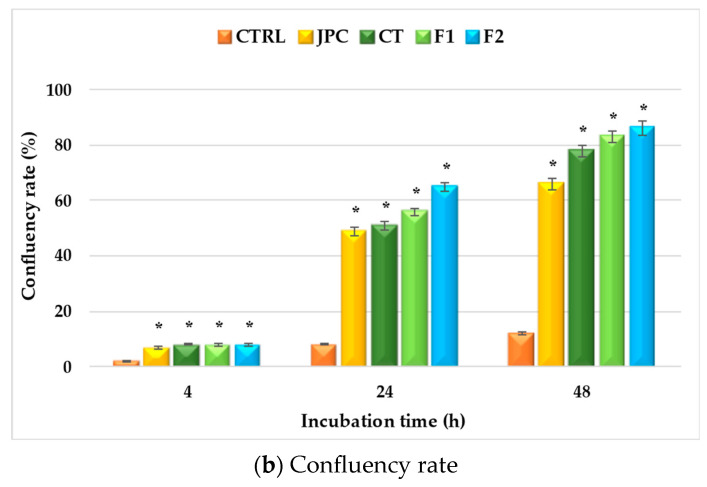
Results for the scratch test: (**a**) Study of the scratch test on monolayers of fibroblasts from the A31 clone of the BALB/3T3 line using JPC, CT, F1 (JPC-CT-ALG 10%), and F2 (JPC-CT-ALG 20%). CTRL represents untreated cells (CONTROL). Representative micrographs (4_ magnification) of treated and control monolayers. (**b**) Confluency rate, expressed as the percentage of scratch closure relative to the initial scratch area, was calculated for BALB/3T3 clone A31 cells. Statistical significance was marked with * for *p* < 0.05 for all tested samples compared with the control.

The scratch assay was performed on BALB/3T3 clone A31 fibroblast cells. This test is typically used to simulate wound closure in vitro, as it follows the healing of a mechanically induced disruption in a cell monolayer. During this process, cell migration at the wound margins and regeneration through cell proliferation can be monitored [72,73]. In the present study, the scratch was created using a pipette tip to mimic an incision-like lesion. To evaluate fibroblast migration in the presence of JPC collagen, CT chitosan, and the F1 and F2 biocomposites, a confluent cell monolayer was used, and wound closure was monitored at 4 h, 24 h, and 48 h, as presented in Figure 11a.

We find that all samples recorded a fairly rapid cell migration and a confluence rate that we present in Figure 11b. After 48 h the percentage of confluence rate reached values of 83% and 86% in the case of the new formulations F1 and F2.

#### 2.7.2. Biological Evaluations in the Wound-Healing Process on HaCaT Cells

**Figure 12:**
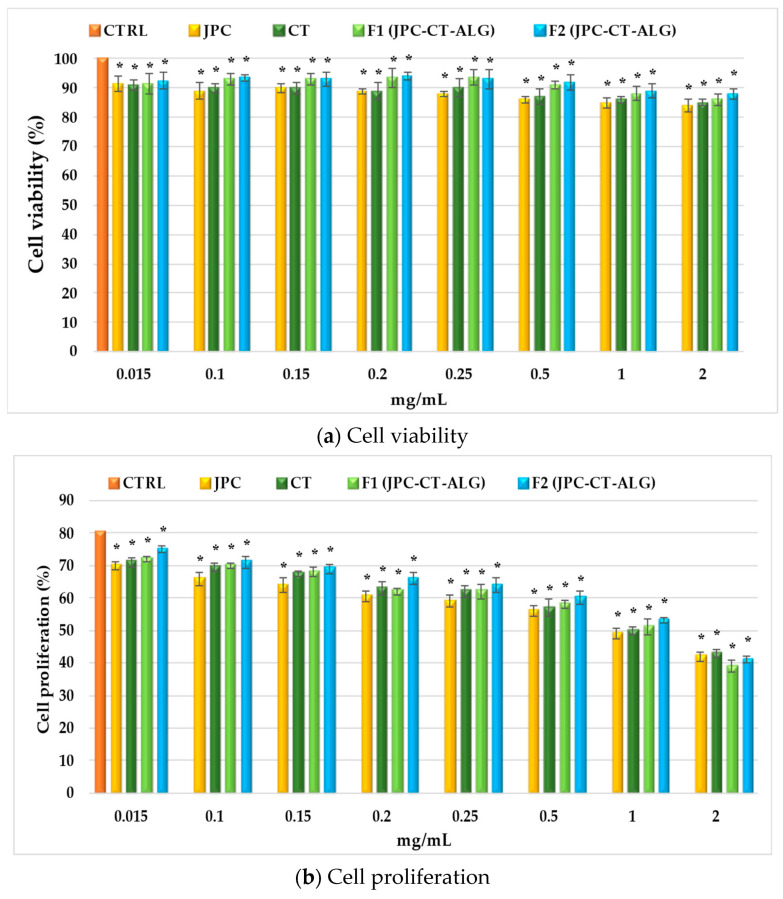
Shows cytotoxicity testing performed on HaCaT cell lines exposed to JPC, CT, and the two biocomposites, F1 (JPC-CT-ALG 10%) and F2 (JPC-CT-ALG 20%), at concentrations ranging from 0.015 mg/mL to 2 mg/mL; (**a**) the results after 24 h of exposure for cell viability; (**b**) the results after 48 h of exposure for cell proliferation. Untreated cells were used as the control. Each result represents the mean ± SD of eight replicates. Statistical significance compared with the control was marked with * for *p* < 0.05.

First, the cytotoxicity of the JPC, CT, F1, and F2 preparations was evaluated on keratinocytes using HaCaT cell lines, and the results are presented in Figure 12a,b. After 24 h of incubation, no obvious cytotoxic effects were observed for any of the tested samples. Cell viability remained high, with values of 86% and 88% recorded for the new F1 and F2 formulations, respectively. After 48 h of incubation, cell proliferation decreased as the sample concentration increased, as shown in Figure 12a,b. For the new F1 and F2 formulations, cell proliferation values of approximately 39% and 40% were recorded at the highest tested concentrations. Statistical analysis using one-way ANOVA followed by the *t*-test showed that all results were statistically significant compared with the control, represented by untreated cells at *p* < 0.05. Statistical significance was marked with the symbol *. At 48 h, the proliferation of HaCaT cells exposed to F1 and F2 decreased to 39% and 41%, respectively, at a concentration of 2 mg/mL.

**Figure 13:**
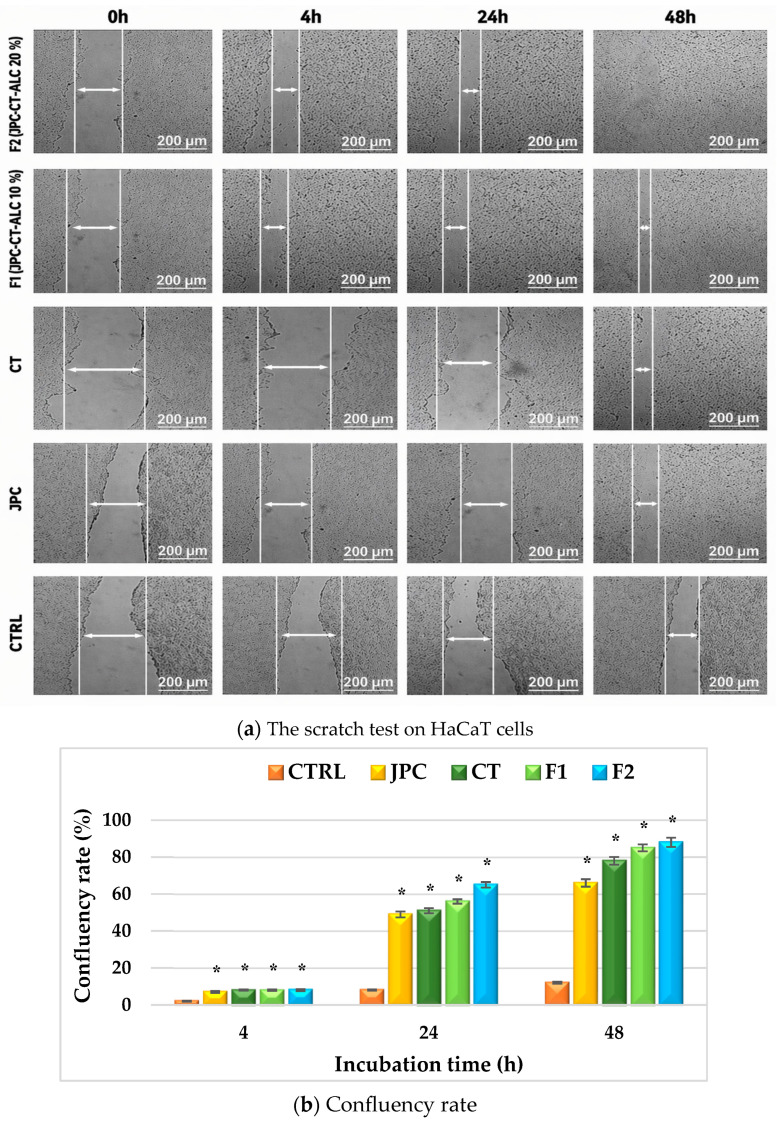
Study of the scratch Ha CaT cells: (**a**) Representative microfilms for scratch test for treated and untreated HaCaT cells; (**b**) confluency rate for all samples made on HaCaT cells. It is marked by the symbol * statistical significance for *p* < 0.05 for all samples taken in work compared to the control.

In the case of HaCaT cells, the scratch test is shown in Figure 13. Keratocyte migration can be observed using a monocamera that monitors healing capacity for 48 h. After 48 h, the scratch area was markedly reduced in the presence of F1 and was almost completely closed in the presence of F2. These observations suggest that the formulations support HaCaT keratinocyte migration and monolayer closure in vitro.

In Figure 13b, the confluence rate, calculated as percentage of scratch closure in respect to initial scratch area, is shown. Statistical significances for *p* < 0.05 for all samples compared to the control are marked with the symbol *.

At 48 h, the scratch closure/confluence rate reached 85% and 88% for F1 and F2, respectively. These results support the wound-relevant activity of the formulations in an in vitro keratinocyte model. Collagen formulations show good biocompatibility with positive effects in their use in wound healing [24,40,71,74]. Individual seaweed extracts have also been studied for their use in wound healing processes [68,69,70].

## 3. Discussions

Wound healing is a process that can be done successfully if the mechanism and biological stages of wound healing are understood.

### 3.1. Biological Mechanisms in Acute Wound Healing

**Figure 14:**
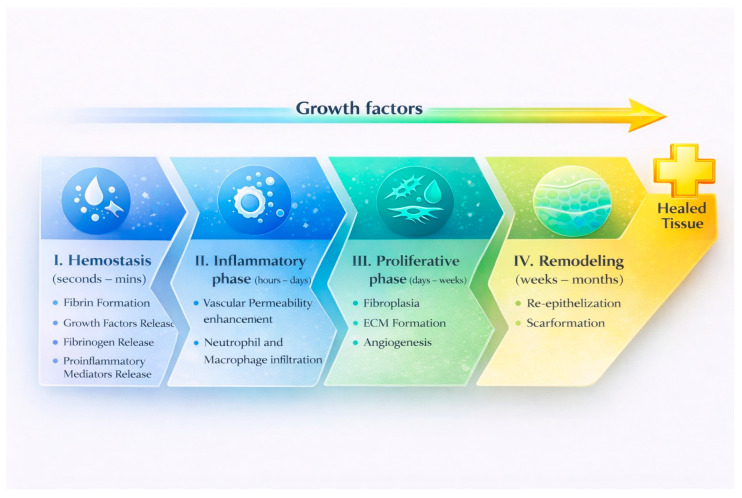
Normal stages in the wound healing process. This figure was created using an artificial intelligence (AI) image generation tool and subsequently edited by the authors.

To promote the healing process, we must analyze the biological processes that occur at each stage of acute wound healing to understand how to act. [5]. These biological processes occur in four stages of acute wound healing and are presented in Figure 14.

The first phase, known as hemostasis, begins immediately after injury. Under the influence of growth factors and pro-inflammatory mediators, fibrinogen is converted into fibrin, which contributes to clot formation and helps limit bleeding. Platelets are also directly involved in clot formation [75]. Their aggregation leads to the release of several chemotactic factors, such as TGF-β1, TGF-β2, and PDGF. These factors recruit inflammatory cells, such as neutrophils, leukocytes, and macrophages, to the wound site, helping to protect the damaged tissue from infection. At the same time, vasoconstriction reduces blood flow through arteriolar narrowing, and within a few minutes, local tissue hypoxia and acidosis may occur [17]. Fibrin not only contributes to clot formation, but also helps limit microbial development and serves as a temporary protective barrier for the injured tissue [76].

The second stage, inflammation, begins shortly after injury and is characterized by the rapid recruitment and activation of immune cells at the wound site. Neutrophils are among the first cells to arrive, guided by signals released from resident cells, serum components, platelets, and nearby blood vessels. Their activation is influenced by complement system activity, platelet degranulation, and bacterial degradation products [76]. Approximately 1–2 h after injury, neutrophils migrate through the endothelial layer of capillary walls and are activated at the wound site by pro-inflammatory cytokines, including IL-1β, IL-6, TNF-α and IFN-γ [77,78,79]. Once activated, neutrophils release antimicrobial and inflammatory mediators, such as cationic peptides, ROS species and proteases [80]. As active blood leukocytes, neutrophils contribute to wound debridement and provide antimicrobial defense [81]. Foreign epitopes, including lipopolysaccharides (LPSs) from invading microorganisms, may further amplify this response [82].

As the inflammatory phase advances, macrophages remove apoptotic neutrophils through phagocytosis, while other neutrophils undergo apoptosis or are eliminated from the wound surface [83]. Macrophages play a central role in this stage because they release several growth factors involved in tissue repair, including FGF, TGF-α, TGF-β, PDGF and VEGF. About 2–3 days after the injury occurs, all of these factors typically reach their peak concentrations and contribute to the formation of granulation tissue, stimulate angiogenesis, and regulate the inflammatory response [83]. Overall, the hemostatic and inflammatory events dominate the early wound-healing response, particularly during the first 2–3 days after injury. The proliferative phase typically lasts from day 4 to approximately day 30 after injury. During this stage, several key repair processes take place, including extracellular matrix (ECM) formation, angiogenesis, and epithelialization. Under the influence of platelet-derived growth factor (PDGF), fibroblasts are stimulated to produce proteoglycans and collagen, marking the beginning of new ECM formation [4]. This matrix includes fibronectin, proteoglycans, glycosaminoglycans, thrombospondins, vitronectin, tenascin, and different collagen types [84].

Angiogenesis also occurs during this phase, allowing the formation of new blood vessels and the repair of damaged vascular structures. This process is sustained by several growth factors released by platelets, including TGF-β, PDGF, and FGF [83]. TGF-β also contributes directly to tissue repair. As a result, granulation tissue develops, which is typically highly vascularized [85]. At the same time, fibroblasts proliferate and migrate into the wound area. Epithelialization begins within hours after injury, starting from the wound margins. Initially, a single layer of epithelial cells covers the wound surface; later, through mitotic division, epithelialization progresses from the edges towards the center. Once epithelial cells from opposite sides meet, migration stops, and the basement membrane begins to form [86]. Remodeling represents the fourth stage of acute wound healing, as shown in Figure 14. This phase follows proliferation and involves the gradual replacement of the fibrin clot with collagen-rich granulation tissue and newly formed blood vessels, ultimately leading to scar formation [87,88]. During remodeling, wound tensile strength increases, while epithelialization and ECM organization continue to mature. Growth factors with polypeptide structures play an important role in this stage by stimulating cell proliferation, supporting the formation of new cells, and contributing to tissue remodeling [31,87]. The remodeling process may last from several weeks to several months and, in some cases, can continue for a year or longer after injury [1,89].

Treatment of acute wounds depends on several factors, including wound location, type, depth, and the presence of infection or foreign bodies. In infected or contaminated wounds, surgical debridement and antimicrobial therapy may be required [81,89].

### 3.2. Characteristics of Chronic Wounds

In chronic wounds, the healing process is usually interrupted or prolonged in one of its normal phases, most commonly the inflammatory phase [90]. When this occurs, the wound becomes more vulnerable to rapid colonization by bacteria and fungi. This microbial burden can reduce the availability of growth factors and promote fibrin degradation, both of which are essential for adequate tissue repair [83]. For this reason, controlling bacterial infection is an important step in improving the healing process [91].

Chronic wounds are also characterized by reduced mitotic activity, impaired growth factor signaling, and decreased fibroblast function [90,92]. In these wounds, persistent inflammation is primarily maintained by neutrophils, partly because macrophages have a reduced capacity to phagocytose and clear cellular debris [9]. At the same time, excessive levels of reactive oxygen species (ROS) can further damage the tissue by increasing oxidative stress within the wound environment. High ROS levels also stimulate the activity of matrix metalloproteinases (MMPs), which can lead to excessive degradation of extracellular matrix (ECM) components and interfere with normal cell migration [93,94]. The resulting ECM damage, together with poor fibroblast activity and an inadequate growth factor response, contributes to defective granulation tissue formation [93].

Several types of chronic wounds, including diabetic ulcers, venous ulcers, and arterial ulcers, are frequently associated with advanced age, reduced mobility, systemic disorders, impaired blood circulation, and metabolic diseases such as diabetes mellitus [85,86,88]. Therefore, the management of chronic wounds should not focus only on the local lesion, but must also address the underlying condition that contributed to wound development [93,95,96].

### 3.3. Microbiological Characteristics of Chronic Wounds

Wounds are frequently exposed to microbial contamination, particularly by bacteria. However, the presence of bacteria does not always have the same effect on healing. Some species can delay or impair tissue repair, whereas others may be part of the normal skin microbiota and do not necessarily cause harm [97]. For this reason, when evaluating wound healing, it is important to differentiate between contamination, colonization, and infection. Wound contamination is common, especially in chronic wounds, and may involve either saprophytic bacteria from the patient’s own skin flora or microorganisms originating from the external environment. Colonization occurs when bacteria multiply within the wound, but without producing a clear tissue-invasive response or obvious clinical signs of infection [97,98,99]. Several microorganisms normally present on the skin surface may be involved in this process, including *Staphylococcus epidermidis*, other *Staphylococcus* species, *Corynebacterium* spp., *Brevibacterium* spp., *Propionibacterium acnes*, and *Pityrosporum* spp. [97].

Infection develops when microorganisms invade the wound surface and surrounding healthy tissue, leading to a harmful interaction with the host. Among the most commonly reported pathogens in wound infections are *Escherichia coli* (*E. coli*), *Klebsiella* spp., *Staphylococcus aureus* (*S. aureus*), *Proteus* spp., *Acinetobacter* spp., *Pseudomonas* spp., and beta-hemolytic streptococci [100]. In early infected acute wounds, the microorganisms usually originate from the normal skin microflora, with beta-hemolytic *Streptococcus* and *S. aureus* being among the most frequent. These bacteria are also commonly detected in diabetic foot ulcers [101,102,103].

As the wound becomes chronic, its microbial profile often changes. Aerobic and anaerobic Gram-negative bacteria, such as *Pseudomonas*, *Proteus*, *E. coli*, *Klebsiella*, and *Clostridium perfringens*, may become more prominent. Gram-positive anaerobic bacteria, including *Clostridium* spp., can also contribute to the infectious process [97].

### 3.4. Parameters That Influence Wound Healing Conditions

Risk factors that may interfere with the wound-healing process include the following [104]:

- Drug treatments that alter the inflammatory response, such as corticosteroids. After oral administration, steroids may reduce cytokine levels and, consequently, decrease collagen synthesis [82,86].
- Chronic diseases, particularly diabetes mellitus, which can significantly impair normal wound repair [95,105,106,107].
- Advanced age, as elderly patients have a higher risk of chronic disease and often show slower inflammatory, migratory, and proliferative responses during healing [81,96].
- Poor nutritional status, especially diets low in proteins and carbohydrates, which can delay wound repair. The formation of granulation tissue depends strongly on adequate nutrition, sometimes even more than on the type of dressing applied [106,107,108,109].
- Inadequate local wound care, including the improper use of dressings or topical preparations. Clean dressings, together with emollient and antibacterial formulations, help maintain a warm, moist, and non-toxic environment that supports natural wound healing [110,111,112].

### 3.5. The Contribution of Marine Ingredients and New Biocomposite Formulations from Natural Resources

The application of treatments with natural compounds in wound healing represents an alternative method that is gaining increasing importance compared to synthetically obtained drug formulations [38,58,113,114].

### 3.6. Structures of Bioactive Compounds of Interest Extracted from Marine Resources

**Figure 15:**
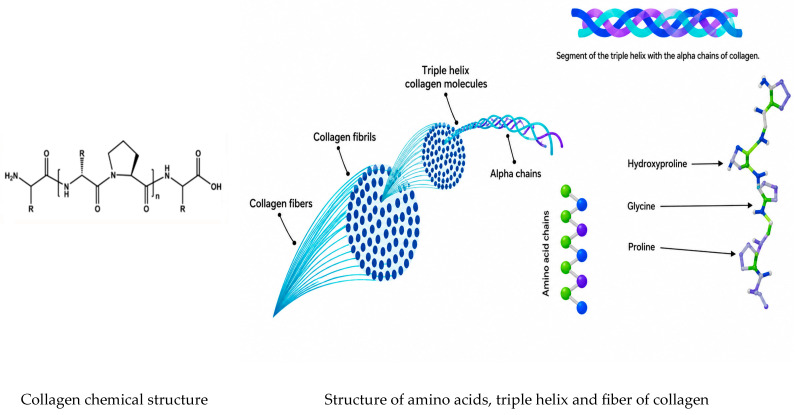
Molecular structure of collagen [31].

The molecular structure of marine collagen consists of collagen fibers and fibrils, and the collagen molecule is a triple helix si sunt redate in Figure 15 [31,56].

**Figure 16:**
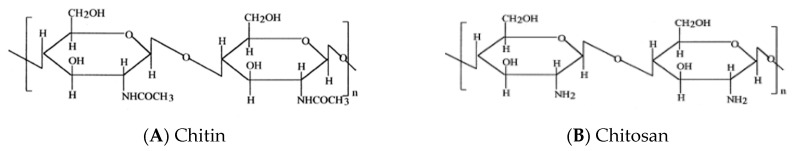
Structure of chitin and chitosan obtained from stone crabs [47].

The molecular structures of chitin and chitosan derived from Black Sea stone crabs are presented in Figure 16.

**Figure 17:**
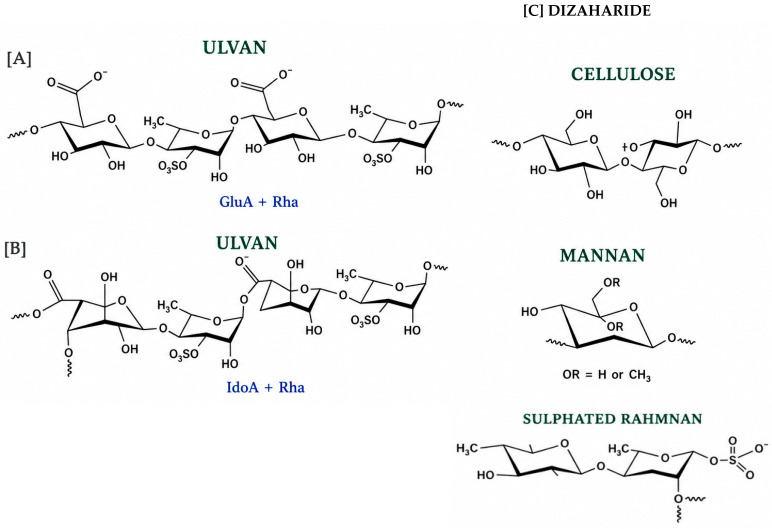
Structure of carbohydrate compounds from the green alga *Cladophora vagabunda*: (**A**,**B**) polysaccharides, including ulvan; (**C**) cellulose and sulfated rhamnan; and monosaccharides, including mannan [53].

Polysaccharides extracted from the green alga *C. vagabunda* are presented in Figure 17, [53]. Green marine algae contain polysaccharides with specific structural features, such as ulvan and other polysaccharides, including cellulose, mannan, and sulfated rhamnan.

The use of collagen in wound healing treatments has a well-founded scientific support. Zheng et al. reported marine bioactive collagen peptides as important agents for wound healing [115]. The collagen used in the preparation of the new F1 and F2 formulations was extracted from the Black Sea jellyfish *R. pulmo*. Structural data obtained by SDS-PAGE, circular dichroism, and FT-IR analyzes indicate that this material corresponds to type I collagen.

In previous studies published by us, we investigated validation parameters for the method of collagen extraction from jellyfish, based on spectrophotometric determinations of the hydroxyproline content in the collagenous material extracted following reaction with Ehrlich’s reagent [56]. We also analyzed a series of physico-chemical characteristics of the collagen extracted from *R. pulmo*, including appearance, color, pH value, and denaturation temperature [56]. Spectrophotometric analysis of the collagen extract was used to identify and quantify the hydroxyproline present in type I collagen obtained from the jellyfish *R. pulmo* [56]. Our results were also confirmed by Addad et al. in their studies on type I collagen from the jellyfish *R. pulmo*. [33].

The relevance of marine collagen for wound-healing applications has also been reported by several other authors [116,117,118]. In particular, jellyfish collagen has been described as a promising material for accelerating wound repair and improving delayed wound closure in diabetic mouse models [119,120].

**Figure 18:**
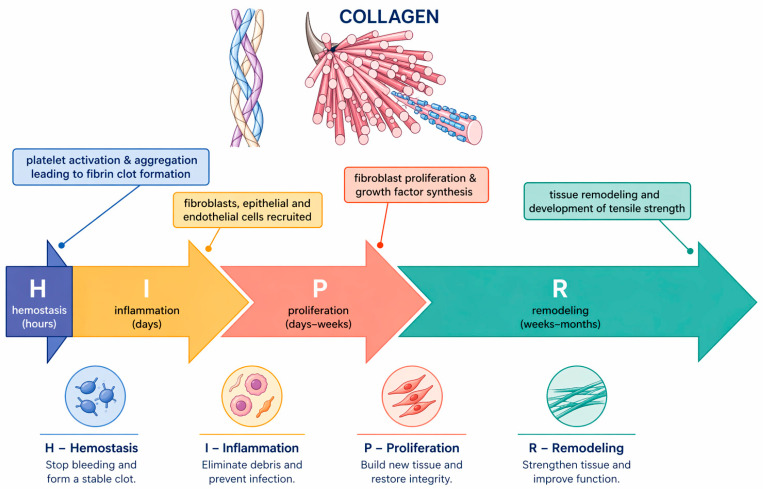
The implications of collagen in all phases of the healing process. H—hemostasis; I—inflammation; P—proliferation; R—remodeling. This figure was created using an artificial intelligence (AI) image generation tool and subsequently edited by the authors.

Sumiyoshi et al. suggested that jellyfish collagen may be suitable for external applications, partly because it denatures rapidly at body temperature. This property could help reduce the risk of unexpected immune reactions that may be associated with the use of invertebrate collagen [119,120]. Collagen participates in all phases of healing as shown in Figure 18 [27].

In another study, Coman et al. used collagen scaffolds incubated with adult human dermal fibroblasts and evaluated their role in wound healing, particularly in relation to moisture control at the wound site [121].

In this biocomposite, the main contribution of protein-based bioactive compounds, including amino acids and collagen peptides, comes from *R. pulmo* collagen collected from the Black Sea. Its composition is comparable to collagen extracted from jellyfish collected from other marine areas, such as the Mediterranean Sea and the Goa coast of India [34,37]. The protein fraction was mainly provided by *R. pulmo* extracts, which contained 61.25 ± 1.25% protein, including 57.25 ± 0.25% collagen in the individual jellyfish extract. This was complemented by the protein content of the green alga *C. vagabunda*, which reached 15.07 ± 0.51%.

Proteins, particularly amino acids, together with polysaccharides and other bioactive compounds such as phenolics and flavonoids, are known to play important roles in wound-healing mechanisms [40]. In the JPC extract, 16 amino acids were identified, including 13 essential and 3 non-essential amino acids.

The polysaccharide fraction of the proposed biocomposite is provided by both chitosan extracted from Black Sea crabs and polysaccharides obtained from the green alga *C. vagabunda*. A small amount of carbohydrates was also detected in the collagen peptide extract, although at a much lower level, 0.65 ± 1.28%, compared with the carbohydrate content of *C. vagabunda*, which reached 62.22 ± 1.34%.

The presence of phenolic and flavonoid compounds was also confirmed. In *C. vagabunda*, the total phenolic content was 405.5 ± 1.21 mg GAE/100 g d.w., whereas the collagen extract showed a considerably lower value of 1.54 ± 0.29 mg GAE/100 g d.w. The total flavonoid content of *C. vagabunda* was 15.6 ± 1.78 mg EC/100 g d.w., while flavonoids were not detected in the collagen extract. These compounds are significant because phenolics and flavonoids are known to contribute to the antioxidant and antimicrobial activity of plant and algae derived materials.

HPLC analysis further confirmed the presence of individual polyphenols. Several polyphenolic compounds were identified in *C. vagabunda*, while only three were detected in the jellyfish collagen extract. A similar pattern was reported by Pesterău et al. for biocomposites based on collagen and brown algae [38]. In addition, Cadar et al. investigated the composition of marine algae and emphasized the value of these resources due to their antioxidant, antimicrobial, and anti-inflammatory properties, which may be beneficial in wound-healing treatments [122,123,124].

The polysaccharide component of the proposed biocomposite is represented by marine chitosan isolated from Black Sea crabs. One of the most relevant parameters of the chitosan used in this study is its molecular mass, determined as 9.05 × 10^5^ g/mol, a value closely related to the degree of deacetylation and comparable with previously reported literature data [47]. The degree of deacetylation reflects the amount of free amino groups present along the polymer chain, which confer a positive charge to chitosan and enable electrostatic interactions with negatively charged molecules. Together with hydroxyl groups, these functionalities contribute to the reactivity, versatility, and solubility of chitosan in acidic aqueous media [125,126,127,128,129,130].

**Figure 19:**
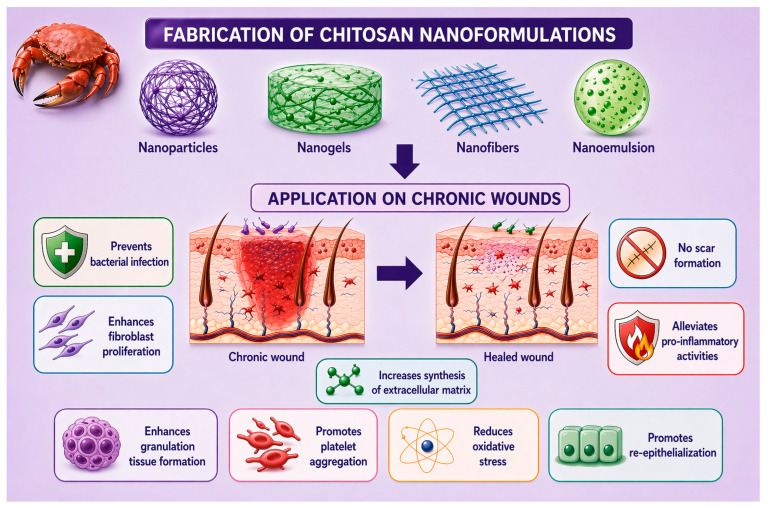
Chitosan applications in chronic wound healing and its advantages. This figure was created using an artificial intelligence (AI) image generation tool and subsequently edited by the authors.

Chitosan is considered a suitable material for wound-healing applications because of its biocompatibility with human skin, its ability to support tissue regeneration, and its hemostatic properties [126]. Chitin and chitosan are also involved in several biological processes associated with wound repair, including leukocyte recruitment, stimulation of granulation tissue formation, accelerated epithelialization, and the release of biologically active mediators such as prostaglandins [127]. In addition, these polymers enhance platelet aggregation and promote the release of platelet-derived growth factors, contributing to the activity of endothelial cells and fibroblasts within the wound environment [128]. Although chitin and chitosan do not directly stimulate endothelial cell or fibroblast proliferation in vitro, their degradation products may induce endothelial cell migration, suggesting an important role in the inflammatory and subsequent repair phases of wound healing [128,129] Figure 19 summarizes the main uses of chitosan and its applications in chronic wound management.

Its biocompatibility, biodegradability, antimi-crobial activity, and immunomodulatory effects are among the main characteristics supporting its use in regenerative medicine [130].

Based on the three marine-derived ingredients, two biocomposite formulations were developed for wound-healing applications: F1 (JPC-CT-ALG 10%) and F2 (JPC-CT-ALG 20%). The visual, rheological, and microbiological analyzes indicated good formulation homogeneity, particularly after 24 h. Rheological evaluation of the JPC-CT-ALG biocomposites showed non-Newtonian behavior, consistent with the Ostwald–de Waele model, suggesting that the formulations may maintain adequate stability over time.

The antioxidant activity, evaluated using the DPPH and reducing power assays, showed that the F1 and F2 biocomposites performed better than the individual ingredients. This effect is applicable in wound healing, where antioxidant activity can help reduce oxidative stress in the wound environment. Similar antioxidant effects have been reported for the individual components in previous studies. For collagen from *R. pulmo*, several recent studies have described its antioxidant potential [34,37,38,131]. Natural products have also been reported to reduced oxidative stress and contribute to skin protection [132,133]. In the case of marine algae, including *C. vagabunda*, antioxidant activity has been associated with both polysaccharides and phenolic and flavonoid compounds [50,55].

In the DPPH assay, at a concentration of 400 mg/mL, the antioxidant activity reached 61.5% for F1 and 68.5% for F2, compared with 27.6% for JPC, 43.3% for CT, and 55.3% for ALG. The reducing power assay showed a similar trend. At 800 μg/mL, the absorbance at 700 nm was 0.75 μg/mg AAE for F1 and 0.88 μg/mg AAE for F2, compared with 0.521 μg/mg AAE for JPC, 0.65 μg/mg AAE for CT, and 0.7 μg/mg AAE for ALG. Ascorbic acid was used as the control and showed a reducing power of 1.962 μg/mg AAE at the same concentration.

Microbial activity is also highly relevant in wound healing, since wounds are frequently exposed to microorganisms and may undergo contamination, colonization, and infection [97,98,100,105]. Therefore, the antimicrobial properties of the developed formulations are important for evaluating their potential use in wound management.

The antimicrobial results showed that the highest inhibition zones were recorded against *S. aureus*. For this strain, the two biocomposite formulations produced inhibition zones of 17.7 mm for F1 and 18.2 mm for F2, compared with 8.5 mm for JPC, 8.9 mm for CT, and 16.5 mm for ALG. A strong antimicrobial effect was also observed against *E. coli*, with inhibition zones of 16.2 mm for F1 and 17.5 mm for F2, while the individual ingredients showed lower values: 7.8 mm for JPC, 9.4 mm for CT, and 15.3 mm for ALG.

The weakest antimicrobial response was observed against *P. mirabilis*, with inhibition zones of 9.2 mm for F1 and 11.3 mm for F2. Among the individual ingredients, the lowest values were recorded for JPC against *K. pneumoniae* and *P. mirabilis*, with inhibition zones of 5.2 mm and 5.6 mm, respectively. The minimum inhibitory concentration (MIC) values obtained for the green algae extracts further supported these findings. The most sensitive strains were *S. aureus* and *E. coli*, both showing the lowest MIC value, 25 µg/mL. *S. epidermidis*, and *K. pneumoniae* showed MIC values of 50 µg/mL, whereas *P. mirabilis* was the least sensitive strain, with an MIC value of up to 100 µg/mL. The higher sensitivity of Gram-positive bacteria, particularly *S. aureus*, may be related to the structure of their cell wall, which contains a thick peptidoglycan layer but lacks the protective outer membrane found in Gram-negative bacteria. This structure may permit easier interaction with bioactive compounds from the marine ingredients and from the F1 and F2 biocomposites. Compounds such as polyphenols and peptides can affect membrane integrity and interfere with essential cellular processes, including protein synthesis. By contrast, Gram-negative bacteria such as *K. pneumoniae* and *P. mirabilis* possess a more complex outer membrane rich in lipopolysaccharides, which can act as a barrier against antimicrobial agents and may explain their lower sensitivity to the tested formulations. Similar antimicrobial effects of chitosan have been reported by Sirbu and Raina et al. [47,126]. Antimicrobial activity has also been described for chitosan obtained from *Pachygrapsus ormoratus* crab shells and for the green alga *C. vagabunda* collected from the Black Sea [47,50].

The in vitro biological activity of the new biocomposite was evaluated using BALB/3T3 clone A31 fibroblast cells and HaCaT keratinocytes. Cytotoxicity testing showed no major cytotoxic effects after 24 h in either cell line, although a significant decrease in cell proliferation was observed after 48 h. The scratch assays performed on BALB/3T3 fibroblasts and HaCaT keratinocytes indicated that the two biocomposite formulations, F1 and F2, supported monolayer closure in vitro. These findings suggest that the formulations may influence cellular processes relevant to wound repair, such as fibroblast and keratinocyte migration and/or proliferation. These beneficial results should be interpreted as relevant preliminary evidence. In future studies, we aim to extend this investigation to include animal models to assess the composite’s biocompatibility, safety, degradation behavior, and efficacy in promoting wound closure and tissue regeneration in vivo. These studies are essential for validating the applicability and supporting further clinical development of this biomaterial, with applications in the treatment of normal, infected, and diabetic wound models.

## 4. Materials and Methods

The most important bioresources used to develop the new pharmaceutical systems for tissue repair applications were the green alga *C. vagabunda,* the jellyfish *R. pulmo*, and the crab *Pachygrapsus mormoratus.* All three species were collected from the Romanian Black Sea coast. Jellyfish and crabs were harvested from the marine waters near Mamaia turist center and the port area of Constanța, while the algal material was collected from the southern part of the coast, particularly from the Costinești resort area.

### 4.1. Chemical Reagents

All reagents used in the study were of analytical grade and were selected according to the requirements of each experimental procedure. Reagents for SDS-PAGE analysis were purchased from Bio-Rad Laboratories (Hercules, CA, USA), while glacial acetic acid (99–100%) was obtained from Merck KGaA (Darmstadt, Germany). The materials used for the antimicrobial assays were provided by the Sanitary Veterinary and Food Safety Directorate of Constanța, Romania.

HaCaT keratinocyte cell lines were purchased from Cell Systems GmbH, Germany, and BALB/3T3 clone A31 fibroblasts (CCL-163) were obtained from ATCC, USA. Dulbecco’s Modified Eagle Medium (DMEM), used for cell activation, was supplemented with 1% penicillin–streptomycin and 10% fetal bovine serum, all supplied by Thermo Fisher Scientific (Waltham, MA, USA). The remaining standards and reagents required for the analysis were purchased from Sigma-Aldrich, Darmstadt, Germany. Experimental controls were acquired separately for each type of analysis performed.

### 4.2. Extraction of Compounds from Marine Sources

#### 4.2.1. *Cladophora vagabunda* Seaweed Extraction

The *C. vagabunda* algal material was collected and transported in new plastic bags filled with seawater, in order to limit water loss through evaporation. To remove salts and adhering impurities, the algae were thoroughly washed, first with drinking water and then with double-distilled water. After cleaning, the biomass was dried in a dark, well-protected area and then ground into powder using an electric mixer. The resulting powder was sieved through a 3 mm mesh sieve to obtain a more uniform particle size.

For the extraction process, two hydroalcoholic ethanol solutions, 70% and 90% (*v*/*v*), were used. The algal powder was macerated in solvent at a solid-to-liquid ratio of 1:10 (*w*/*v*) for 24 h at room temperature, approximately 24 °C. The extraction was carried out in hermetically sealed brown glass containers to prevent light-induced degradation, air exposure, and solvent evaporation. During maceration, the mixtures were periodically stirred to improve the transfer of bioactive compounds into the solvent. After extraction, the obtained solutions were concentrated using a rotary evaporator and then filtered three times through Whatman No. 1 filter paper, resulting in a clear green extract. The final extracts were transferred to borosilicate glass containers and stored under refrigeration until further use [133].

#### 4.2.2. Extraction of Collagen and Collagen Peptides from *R. pulmo*

The first step in processing the Black Sea jellyfish *R. pulmo* included cleaning the biological material through successive washing steps. Initially, the jellyfish samples were pretreated with ultrapure water and sodium chloride (NaCl) solutions. Demineralization was then carried out using a 0.5 M ethylenediaminetetraacetic acid sodium salt solution (EDTA-Na).

Collagen extraction was performed according to the protocol described by Cadar et al. (2024) for the isolation of collagen from marine invertebrates [28]. Briefly, fragments of the jellyfish body, including the umbrella and oral arms, were washed and treated with 0.5 M acetic acid for 30 min. Pepsin was then added at a concentration of 10% (*w*/*v*) and the mixture was stirred for three days at 4 °C.

After enzymatic digestion, the suspension was centrifuged at 10,000 rpm for 60 min. The supernatant was collected, and collagen precipitation was induced by adding 2 M NaCl in the presence of 0.05 M Tris buffer at pH 7.0. The resulting precipitate was separated by centrifugation at 20,000 rpm for 60 min and then redissolved in 0.5 M acetic acid. To remove residual acetic acid, the obtained solution was dialyzed against ultrapure water for 2 h. All extraction steps were performed at a constant temperature of 4 °C to maintain collagen stability. To calculate the extraction yield of collagen, the following formula was used:

(1) $$Collagen\;yield\;\left(wet\right)\%=\frac{Weight\;of\;extracted\;collagen\;(g)}{Weight\;of\;wet\;jellyfish\;(g)}\;\times \;100$$

Collagen peptides from *R. pulmo* were obtained using the enzymatic hydrolysis method described by Felician et al. (2019) [24]. Briefly, 1 g of previously extracted collagen was dispersed in 200 mL of ultrapure water and maintained in a water bath at 37 °C. Collagenase type II was then added at 5% (*w*/*w*, enzyme/substrate) and the mixture was continuously stirred for 5 h.

By keeping the sample at 95 °C for 10 min, the enzymatic reaction ceased. After cooling to room temperature, the suspension was centrifuged at 5000 rpm for 30 min. The resulting supernatant, containing the jellyfish collagen peptides (JPCs), was collected. The peptides were either used directly or lyophilized and stored at 4 °C for further use.

#### 4.2.3. Extraction of Chitosan

Chitosan was obtained from crab shells through a chemical extraction procedure involving three main stages: deproteinization, decolorization, and deacetylation. Deproteinization removes proteins and part of the carbohydrate fraction, while decolorization eliminates pigments, mainly carotenoids and other colored compounds. The final and most important phase is deacetylation, through which chitin is converted into chitosan [47,127,134].

Deproteinization. The powdered shell material was treated with 2% KOH solution at a solid-to-liquid ratio of 1:10 (*w*/*v*) under continuous magnetic stirring. After treatment, the solid residue was separated by filtration and repeatedly washed with deionized water until neutral pH was reached. The resulting material was dried overnight at 50 °C [47,128,135,136].

Decolorization. The deproteinized material was treated with acetone to remove pigments, mainly carotenoids and other colored compounds, until the material reached the characteristic natural color of chitin. The solid fraction was filtered through Whatman filter paper and dried again at 50 °C to obtain chitin [47,128].

Deacetylation. The dried chitin was treated with 20% NaOH solution at 50 °C for 1 h under continuous stirring to convert chitin into chitosan. After the reaction, the mixture was filtered, and the recovered solid was washed repeatedly with deionized water until neutral pH was reached. The obtained chitosan was dried at 50 °C and stored in a desiccator until further use [47,127,135].

### 4.3. Development of Novel Pharmaceutical Systems

The biocomposites intended for wound-healing applications were prepared by combining three marine-derived components: collagen, chitosan, and algal extract. First, the collagen extracted from jellyfish was dissolved in distilled water to obtain a 1% gel. In parallel, chitosan, with a molecular weight of 9.05 × 10^5^ g/mol and obtained from crab shell chitin, was dissolved in an acetic acid/sodium acetate buffer (0.2 M/0.1 M) until a homogeneous gel was formed. The two gels were then mixed in a 1:1 (*v*/*v*) ratio to produce a uniform polymer matrix. The algal extract was prepared separately by macerating *Cladophora vagabunda* biomass in 70% ethanol for 10 days, followed by filtration to recover the hydroalcoholic extract. This extract was then added to the collagen–chitosan matrix at two different concentrations, 10% and 20% (*v*/*v*), resulting in two biocomposite formulations, F1 and F2. The final mixtures were homogenized to obtain uniform hydrogels, which were subsequently used for physico-chemical characterization and biological testing.

### 4.4. Phisyco-Chemical Determination for R. pulmo

#### 4.4.1. SDS-PAGE Tehnique

To characterize the protein profile of *R. pulmo* jellyfish collagen, SDS-PAGE analysis was performed. First, the sample was centrifuged at room temperature for 5 min to remove undissolved fractions, after which the obtained material was treated with phosphate buffer solution. The supernatant was mixed for 5 min in equal parts with the sample buffer. Sample preparation was carried out using Laemmli reagent and included dialysis, cell lysis, and protein denaturation at 95 °C. The prepared samples were then loaded onto the polyacrylamide gel and subjected to electrophoresis. The unit ran at 85 V for 30 min, after which it ran at 95 V until completion. After electrophoresis, the gel was first stained and then destained using a mixture of methanol, acetic acid, and distilled water. Protein bands were analyzed using a calibrated GD-800 densitometer (Bio-Rad Laboratories, Hercules, CA, USA), and the data were processed with Quantity One software, version 4.6.3 (Bio-Rad). Similar studies reported in the literature have also used SDS-PAGE for the characterization of jellyfish-derived collagen [33,35,37].

#### 4.4.2. Circular Dichroism Spectral Analysis

In order to determine the structure of proteins, this optical spectrophotometric technique was used, which is based on the differential absorption of radiation, providing relevant information on the structural conformations of proteins. The use of this method was reported by Pesterau et al. (2025) and Pascale et al. (2026) [38,58]. Thus, in the far UV range (λ = 170–240 nm), the secondary structure can be investigated, while in the near UV range (λ = 260–300 nm), tertiary structures are monitored, in particular in the vicinity of aromatic amino acid residues. A Jasco J-810 spectropolarimeter (JASCO Corporation, Tokyo, Japan) was used to perform the determinations, and the UV-CD spectra were recorded in the wavelength range 195–250 nm. The final spectrum was obtained as the average of four measurements performed at 25 °C, consecutively. In the case of collagen extracted from *R. pulmo*, prepared in a 0.5 M acetic acid solution, the CD spectrum revealed a minimum at 198 nm and a maximum at 214.5 nm.

#### 4.4.3. Spectroscopic FT-IR Analysis

FT-IR spectroscopy was performed using a Jasco 4200 FT-IR spectrophotometer operated with Spectra Manager software (JASCO Corporation, Tokyo, Japan). The spectra were recorded over the range of 350–7800 cm^−1^. Before analysis, the samples were solubilized in acetic acid at a 1:1 (*v*/*v*) ratio. For spectral acquisition, the samples were finely ground, homogenized with KBr, and then analyzed in the infrared region. The characteristic spectral bands corresponding to collagen peptides were evaluated in the 700–4000 cm^−1^ range [38].

### 4.5. Analysis of the Biochemical Composition of the Studied Species

The ash and moisture contents of the studied samples were determined according to the AOAC standardized methods, 2019 [137]. Ash content was measured by incinerating the samples in an electric furnace at 500 °C for 4 h. Moisture content was determined by drying 2 g of each sample in an incubator at 105 °C until constant mass was reached [136]. The results were expressed as percentages.

#### 4.5.1. Biochemical Composition for *C. vagabunda*

Total protein and nitrogen determination. The protein content, and implicitly the total nitrogen content, of the algal samples were determined using the Kjeldahl method. The analysis was performed with a UDK DK6 digestion system equipped with a 127 distillation unit and Velp software (Usmate, Italy, https://www.velp.com/). In the first stage, the samples were digested with sulfuric acid in the presence of mercury and selenium, which acted as catalysts. The samples was then alkalized, and the released ammonia was steam-distilled, absorbed in boric acid solution, and titrated with hydrochloric acid. The results were expressed as percentages relative to the amount of algal material used [50,55].

Lipid determination. The lipid content was determined using a slightly adapted version of the method described by Rohani-Ghadikolael [138]. Lipids were extracted from the samples for 5 h using the Soxhlet method, with dichloroethane as the solvent. After solvent removal by evaporation, the total lipid content was determined gravimetrically by analyzing two aliquots of each extract. The values were calculated according to the initial mass of the powder and expressed as percentages [138,139].

Carbohydrate determination. The carbohydrate content was determined using aqueous extracts of the green alga *C. vagabuda*, following a method adapted from Albalasmeh [140]. For the analysis, a 5% aqueous phenol solution was freshly prepared and added to the acidic carbohydrate-containing solution. Quantitative determination was performed spectrophotometrically using a VWR UV-6300PC double-beam spectrophotometer (VWR International, Radnor, PA, USA) at 490 nm. The method is based on the direct linear relationship between the carbohydrate concentration in the sample and the measured absorbance. The values were calculated using a glucose calibration curve and the corresponding equation:

(2) y = 0.1009x−0.0024

where x represents the absorbance and y the carbohydrate concentration, and the correlation coefficient R^2^ is 0.992 [137,140].

Total dietary fiber determination. The total dietary fiber content, together with the soluble and insoluble fractions, was determined according to the methods described by Yaich [141]. Fiber quantification was carried out using two approaches: the Prosky method, based on gravimetric determination, and the Englyst method, which follows an enzymatic-chemical procedure [141]. Among these, the gravimetric method was preferred because of its simpler applicability.

#### 4.5.2. Biochemical Composition for *R. pulmo*

Protein determination. The methods used for the biochemical characterization of jellyfish-derived samples were selected and adapted from the protocols described by Leone. (2015), De Domenico (2019), D’Ambra. (2022), and Migone (2022) [34,35,36,40].

Protein content was determined following the procedure reported by De Domenico et al. (2019), using 96-well round-bottom microplates and an Infinite M200 Quad4 monochromator detection system (Tecan, Männedorf, Switzerland) [35]. Serum albumin was used as the calibration standard, and the results were expressed as the mean of three independent replicates. Proteins represent the main class of compounds in jellyfish biomass, as previously reported in the literature [35,142].

Lipid determination—Lipid content was evaluated using a gravimetric method based on micro-Soxhlet extraction. Briefly, 100 mg of sample was extracted with a chloroform–methanol mixture at a ratio of 2:1 (*v*/*v*) [38]. To remove non-lipid compounds, the crude lipid extract was washed with 0.9% NaCl solution. The solvent was then evaporated at 70 °C, and the remaining residue was kept overnight in a desiccator before weighing for lipid content determination.

Carbohydrates determination—To improve the efficiency of the extraction process, the experimental parameters described by Zhang et al. (2014) were adjusted as follows: extraction time, 4 h; temperature, 100 °C; and raw material-to-water ratio, 1:7.5 [143]. An enzymatic treatment with papain, followed by treatment with Sevag reagent, was applied to remove proteins from the crude extracts. The polysaccharide fraction was then separated by ethanol precipitation using different ethanol concentrations, namely 60%, 70%, and 80% [40,143].

#### 4.5.3. Biochemical Composition of Chitosan

Solubility determination. Chitosan solubility was determined using centrifuge tubes of known mass, into which chitosan powder dissolved in 1% acetic acid solution was added [47,134]. The resulting suspensions were first centrifuged at room temperature for 30 min at 250 rpm, followed by a second centrifugation step for 10 min at 10,000 rpm.

After decanting the supernatant, the undissolved particles were washed with distilled water, and the centrifugation step was repeated. The remaining residue was then dried in an oven at 60 °C for 24 h. After drying, the percentage of chitosan solubility was calculated using the equation described by Fernandez-Kim (2004) [135]:

(3) $$Solubility\;\%=\frac{\left(m_{initialtube}+CT\right)-(m_{finaltube}+CT)}{\left(m_{initialtube}+CT\right)-\;m_{initialtube}}\times \;100$$

pH determination—The pH values for the obtained chitosan solutions were determined using a Consort C861 multi-parameter analyzer pH meter, equipped with an SP10B type electrode, and the results were recorded accordingly.

For this analysis, the chitosan solution was prepared by adding 0.2 g of dry chitosan powder to an aqueous solution of 0.1 M HCl, with continuous magnetic stirring at 50 °C for 30 min. After this step, an additional 25 mL of distilled water was added, and stirring was continued until a homogeneous solution was obtained, confirming complete dissolution of chitosan. The degree of deacetylation was determined by titrating the prepared chitosan solution with standardized NaOH solution. At the end of the titration, the volume of NaOH consumed for the neutralization of protonated amino groups was recorded. The degree of deacetylation was then calculated using the following equation:

(4) $$DD\;\%=2.03\frac{V_{2}-V_{1}}{m+0.0042(V_{2}-V_{1})}$$

where, V_2_,V_1_ represent the volumes of NaOH corresponding to the inflection points, and m is the mass of the sample. The coefficient 2.03 is derived from the molecular weight of chitin, and the coefficient 0.0042 is derived from the difference between the molecular weights of chitosan and chitin [47,134].

Molecular weight determination—Chitosan powder was dissolved in a 0.2 M acetic acid/0.1 M sodium acetate solution using a magnetic stirrer and hot plate at 50 °C. The molecular weight of chitosan was determined by viscometric measurements of chitosan solutions. An Ubbelohde capillary viscometer (SCHOTT, Mainz Instruments, Germany), equipped with the Viscoclock module for recording flow times, was used. The molecular weight was calculated using the Mark–Houwink equation:

(5) $$Ƞ=kM^{a}$$

where, Ƞ represents the intrinsic viscosity, M is the molecular weight average viscosity, and a and k are two constants, which have the following values 0.96 and 1.424 × 10^−5^ (dL/g), respectively [47,135].

#### 4.5.4. Determination of Amino Acid Content from *R. pulmo* Collagen

The amino acid content of jellyfish collagen was analyzed by HPLC (type 626 LC System, Waters, Milford, MA, USA) equipped with a 474 fluorescence detector, a 717 Plus autosampler, and Millennium Chromatography Manager programs. For sample preparation, 0.2 g of lyophilized jellyfish material was mixed with 0.75 mL of trifluoroacetic acid, 5 mL of double-distilled water, and 50 μL of internal standard solution, DL-norleucine 5 M in water. The mixture was then incubated for 20 min. After incubation at 4 °C, the sample was centrifuged for 20 min at 3500 rpm. The supernatant was filtered and dried under a stream of nitrogen, and the residue was redissolved in 1 mL of double-distilled water. Amino acids were quantified using the AccQ-Tag protocol (Waters, Milford, MA, USA). Similar methods have been reported for determining the amino acid profile of other jellyfish species [38,144].

### 4.6. Analysis of Polyphenolic Content

#### 4.6.1. Analysis of Total Polyphenolic Content (TPC)

TPC was evaluated for both extracts of the green alga *C. vagabunda* and collagen from the jellyfish *R. pulmo*. The modified Folin-Ciocalteu method was used to identify polyphenols in the green alga [108,145]. Using a VWR UV-VIS double-beam spectrophotometer (VWR International, Radnor, PA, USA), the absorbance was read at a wavelength of 765 nm. The equation on the basis of which the calibration curve was constructed is the following:

(6) y = 0.0078x + 0.1861

The calibration curve showed a correlation coefficient of $R^{2}=0.9959$. The final results were presented as the mean ± SD for *n* = 3 measurements. The values obtained were reported as mg GAE/g d.w. Analogous values for the total polyphenol content of *C. vagabunda* have been reported by Cadar et al. [50].

For the jellyfish samples, total phenolic content was also determined using a slightly modified Folin–Ciocalteu colorimetric method [38]. Briefly, 100 μL of sample was mixed with 500 μL of Na_2_CO_3_ solution and 500 μL of Folin–Ciocalteu reagent. After 2 h of incubation in the dark, the absorbance of the mixture was measured. The standard was gallic acidand the calibration curve was obtained using the following equation:

(7) y = 0.0067x − 0.029

having a correlation coefficient R^2^ = 0.9998. The results are expressed in mg GAE/g d.w.

#### 4.6.2. Analysis of Total Flavonoid Content (TFC)

TFC was obtained using a colorimetric method, with quercetin as the reference standard [146,147]. For the preparation of the quercetin standard solution, 1 g of quercetin was dissolved in methanol and brought to a final volume of 10 mL. Serial dilutions were then prepared to obtain standard concentrations of 25, 50, 75, and 100 μg/mL. For each standard solution, 0.3 mL of 1 M potassium acetate, 0.3 mL of 10% aluminum trichloride solution, 3 mL of methanol, and distilled water were added to a final volume of 10 mL. The mixtures were incubated for 30 min. The absorbance was then measured at 431 nm. To calculate the flavonoid content, the quercetin calibration curve described by the following equation was used:

(8) y = 0.0078x + 0.1842

the correlation coefficient being R^2^ = 0.999. The data obtained were expressed as quercetin equivalents (mg QCE/g seaweed). These results are consistent with studies evidenced in the literature [50].

#### 4.6.3. Determination of Individual Phenol Content

Individual phenolic compounds in the *C. vagabunda* extract and jellyfish collagen extract were determined using the protocol described by Sirbu et al. [55]. HPLC analysis is commonly used for the identification and quantification of individual phenolic compounds. The analysis was performed on an Agilent 1200 HPLC system (Agilent Technologies, Santa Clara, CA, USA), equipped with an autosampler, quaternary pump, detector set at 280 nm and 330 nm, and a column compartment maintained at 35 °C. Peak areas and retention times were automatically recorded using ChemStation software (Agilent version B.07.xx). The mobile phase used for the analysis was prepared from a 1% phosphoric acid solution in water (1 mL phosphoric acid/1000 mL H_2_O). The chromatographic system used is provided with four working channels, and the separation of the compounds was performed using a C18 chromatographic column (5 µm), maintained at a controlled temperature of 35 °C. The analysis was carried out at a flow rate of 1.5 mL/min and an injection volume of 20 µL.

The equipment has four tanks corresponding to the chromatographic phases: cuvettes A and C for the stationary phases, and cuvettes B and D for the mobile phases. The mobile phases were represented by acetonitrile (B) and 0.1% fosforic acid (D), each introduced into 1 L containers. The stationary phases consisted of water (A) and methanol (C), also in 1 L containers. The elution of the compounds was performed in an isocratic gradient regime, using a constant ratio of 10% B and 90% D throughout the analysis.

For the *C. vagabunda* extract, phenolic standards at a concentration of 0.05 mg/mL showed the following retention times: 4-aminobenzoic acid, 3.455 ± 0.005 min; benzoic acid, 9.468 ± 0.098 min; caffeic acid, 8.81 ± 0.07 min; chlorogenic acid, 3.501 ± 0.015 min; ellagic acid, 15.303 ± 0.03 min; ferulic acid, 8.865 ± 0.06 min; gallic acid, 0.990 ± 0.03 min; p-hydroxybenzoic acid, 5.933 ± 0.006 min; protocatechuic acid, 3.130 ± 0.008 min; pyrogallolic acid, 0.910 ± 0.025 min; salicylic acid, 15.952 ± 0.03 min; and vanillic acid, 6.919 ± 0.05 min. These data are comparable with values previously reported in the literature [55].

For the collagen extract, the phenolic standards showed the following retention times: caftaric acid, 0.4697 ± 0.05 min; gallic acid, 3.0278 ± 0.07 min; and syringic acid, 0.1942 ± 0.03 min. Phenolic compounds were identified by comparing the retention times obtained for the samples with those of the corresponding standards [38]. The results obtained for both algal and collagen extracts were expressed as mg/100 g f.w., as mean ± standard deviation, and as percentages.

### 4.7. Physico-Chemical Determinations for JPC-CT-ALG Formulations

#### 4.7.1. Organoleptic Properties

The newly developed JPC-CT-ALG biocomposites were designed as topical hydrogel formulations for wound treatment and tissue repair. The hydrogels were prepared using collagen peptides extracted from *R. pulmo* and chitosan obtained from the crab *Pachygrapsus mormoratus*. Hydroalcoholic extract from the green algae *C. vagabunda* was then incorporated at two concentrations, 10% and 20%, resulting in two distinct formulations. Each formulation was evaluated in terms of appearance, color, and sensory characteristics.

#### 4.7.2. Optical Microscopic Characterization of JPC-CT-ALG Composites

The new JPC-CT-ALG composites were examined by optical microscopy after being applied onto plates in the presence of formalin solution [38]. Observations were performed using a Nikon Eclipse optical microscope equipped with a Nikon Digital Sight DS-FI2 digital camera (Tokyo, Japan). The achieved images were then analyzed and described.

#### 4.7.3. Rheological Analysis of Formulations

Rheological studies were performed on the two formulations, F1 (JPC-CT-ALG 10%) and F2 (JPC-CT-ALG 20%), to evaluate their stability for pharmaceutical use and to characterize the flow behavior of the hydrogels. The measurements were carried out at a controlled temperature of 23 ± 0.1 °C using a Haake VT 550 rheoviscosimeter equipped with a sensor system suitable for medium-viscosity samples and RheoWin 4 software (Thermo Fisher Scientific, Waltham, MA, USA).

The rheograms and flow curves obtained from the measurements were used to identify the rheological model corresponding to each formulation. The rheological behavior was evaluated using Equations (9)–(12), presented in Table 8.

### 4.8. Determination of Antioxidant Activity

#### 4.8.1. DPPH Assay

The method is based on the capacity of antioxidant compounds to neutralize the free radical α-diphenyl-β-picrylhydrazyl (DPPH). The DPPH radical has an initial purple color, which changes to yellow in the presence of antioxidants.

For the test, a 1 M DPPH ethanol solution was prepared. For each sample, 1.5 mL was mixed with 4 mL of the 1 M DPPH ethanol solution. After a 30-min incubation, the absorbances were measured at 517 nm. A UV-VIS spectrophotometer (A. Kruss UV-VIS 6500, Optronic, Germany) was used. The antioxidant capacity was calculated as a percentage using the equation:

(13) $$DPPH\;\left(\%\right)=\frac{A_{control}-A_{sample}}{A_{control}}\;\times \;100$$

where, A_control_ was the absorbance of the control sample, and A_sample_ represents the absorbance of the samples to be analyzed. The positive control was ascorbic acid.

The method was applied both for the algae extract, the collagen and chitosan extract, and for the two new composites. Based on the calibration curves, using nonlinear regression, IC_50_ values were also determined for all samples taken into work, these being expressed as the mean ± standard deviation of three replications.

#### 4.8.2. Reducing Power Test

The reducing power assay is commonly used to evaluate the antioxidant capacity of polyphenolic compounds. The method applied in this study was a slightly modified version of the procedure previously reported by Cadar et al. [50].

In short, 1 mL of each sample was mixed with 2 mL of 1% potassium ferricyanide solution and 2 mL of 0.3 M phosphate buffer. The samples was incubated for 30 min at 55 °C, after which 2 mL of 10% trichloroacetic acid was added. From the resulting solution, a 2 mL aliquot was collected and mixed with 1 mL of 0.1% ferric chloride and 3 mL of distilled water. After a 10-min reaction, the absorbance was measured at 700 nm. A UV-VIS spectrophotometer (A. Kruss UV-VIS 6500, Optronic, Königsbronn Germany) was used. Ascorbic acid was used as the standard. The results were expressed in μg/mg AAE, ascorbic acid equivalents. The results were reported as mean ± standard deviation.

### 4.9. Determination of Antimicrobial Activity

Antimicrobial activity was evaluated for all analyzed samples, including the green alga extract of *C. vagabunda*, the collagen extract, chitosan, and the two newly developed formulations, F1 and F2. The assay was performed using the agar well diffusion method, with slight modifications. Antimicrobial efficacy was assessed by measuring the inhibition zone diameters according to the Kirby–Bauer method [147]. The antimicrobial tests were carried out against a representative panel of microorganisms, including three Gram-negative bacteria, *K. pneumoniae* (ATCC 13883), *E. coli* (ATCC 25322), and *P. mirabilis* (ATCC 25933); two Gram-positive bacteria, *S. aureus* (ATCC 25923) and *S. epidermidis* (ATCC 12228); and one fungal strain, *C. albicans* (ATCC 10231). The culture media, bacterial strains, and fungal strain were provided by the Veterinary and Food Safety Directorate of Constanța, Romania. Chloramphenicol at 5 mg/mL was used as the positive control, while untreated microorganisms were used as the negative control. The strains were cultivated on agar medium at 37 °C. The medium was prepared from 20 g agar, 5 g NaCl, 5 g peptone, and 3 g beef extract dissolved in distilled water. To obtain lawn cultures, the microbial suspensions were uniformly spread over the entire surface of the plates [38,148]. The plates were then incubated for 24 h at 37 °C.

The minimum inhibitory concentration (MIC) was also determined for each analyzed sample and was defined as the lowest extract concentration capable of producing a visible inhibition zone after incubation.

### 4.10. Biological Analysis

#### 4.10.1. Cell Viability and Cell Proliferation of BALB/3T Clone A31 Fibroblasts

Cell viability and proliferation were evaluated using two cell lines: BALB/3T3 clone A31 fibroblasts, obtained from ATCC (New York, NY, USA), and HaCaT keratinocytes, provided by Cell Systems GmbH (Eppelheim, Germany). The cells were maintained in Dulbecco’s Modified Eagle Medium (DMEM) supplemented with 2 mM L-glutamine, 1% penicillin/streptomycin, and 10% bovine calf serum (Merck, Darmstadt, Germany) [74,75].

Both cell types were cultured under standard conditions, in a humidified atmosphere containing 5% CO_2_ at 37 °C. When the cultures reached 80–90% confluence, the cells were subcultured. Before subculturing, the monolayers were washed with PBS, detached using trypsin–EDTA, and resuspended in fresh culture medium, as described in the literature [40]. For the assays, cells were seeded in 96-well plates at different densities, depending on the cell type and incubation time. BALB/3T3 cells were seeded at 1 × 10^4^ or 2.5 × 10^4^ cells/well, while HaCaT cells were seeded at 3 × 10^4^ or 2 × 10^4^ cells/well for the 24 h and 48 h incubation periods. After incubation with the samples, the medium was replaced with fresh medium containing 10% WST-1 reagent, and the cells were incubated for an additional 4 h under the same conditions. Formazan formation was then quantified using a BioTek 800/TS multimode microplate reader (Thermo Scientific, Waltham, MA, USA) at 465 nm, with 655 nm used as the reference wavelength.

#### 4.10.2. Scratch Test on BALB/3T3 Cells and HaCaT Cells

The in vitro scratch assay was performed according to the methods described by Migone et al. (2022) and Fabiano et al. (2021), at the Constanța County Emergency Hospital, Romania [40,149]. BALB/3T3 fibroblasts were seeded in 12-well plates at a density of 1.25 × 10^5^ cells/well in DMEM supplemented with 1% calf serum. After 24 h of incubation in a CO_2_ atmosphere, the cells reached maximum confluence.

HaCaT keratinocytes were seeded under similar conditions, using DMEM supplemented with 1% fetal bovine serum, at the same cell density. In this case, full confluence was reached after 48 h. After confluence, a mechanical scratch was created in each cell monolayer using a sterile 200 μL pipette tip. The wells were then washed three times with PBS to remove detached cells and debris.

After washing, 2 mL of each sample was added to the wells, reaching a final concentration of 0.125 mg/mL. Simple culture medium was used as the control. Images were obtained using a Nikon Eclipse Ts2R inverted microscope equipped with a 4× objective, immediately after scratching, and then after 4, 24, and 48 h. These images were used to monitor the progression of wound closure over time.

The obtained images were analyzed using ImageJ software, version 1.54 (NIH, USA), to determine the cell migration rate [38]. The percentage of wound closure over time was calculated using the following equation:

(14) $$Confluency\;rate\;\left(\%\right)=\;\frac{Area\;T_{0}-Area\;T}{Area\;T_{0}}\;\times \;100$$

where Area T_0_ represents the area at time 0 and Area T is for each sample at the endpoint. The cell migration rate was expressed as a percentage and was defined as the difference between the area covered after treatment and the area of the cell-free area immediately after scratching.

### 4.11. Statistical Analysis

Statistical analysis was performed using parametric tests in SPSS software, version 16.0. Differences between the groups represented by the marine-derived components, seaweed extract, collagen, and chitosan, and the newly developed biocomposites were evaluated by one-way ANOVA analysis (Microsoft Office Professional Plus 2019 (Microsoft Corporation, Redmond, WA, USA)).

In general, the experimental results were expressed as the mean ± standard deviation of three independent determinations. Where a different number of replicates was used, this was specified accordingly. When significant differences were observed, Duncan’s multiple comparison test and Student’s *t*-test for paired comparisons were applied. The statistical significance was set at *p* < 0.05 for all analyses.

## 5. Conclusions

The wound-healing process aims not only to restore tissue integrity, but also to reduce pain, prevent complications, and limit the costs associated with long-term wound care. Current therapeutic directions increasingly focus on innovative treatments based on natural products, which can be used to develop optimized pharmaceutical formulations with improved efficacy, lower toxicity, and improved patient compliance. In this context, the marine environment represents a rich source of bioactive compounds, many of which remain insufficiently explored for therapeutic use.

This study emphasizes the potential value of the jellyfish *R. pulmo*, not as an unwanted marine resource, as it has often been regarded, but as a useful source of collagen peptides with relevance for wound-healing applications. Other important compounds investigated in this work are polysaccharides, obtained from chitosan extracted from stone crabs and from the green alga *C. vagabunda*. Together with collagen peptides and polysaccharides, phenolic and flavonoid compounds from seaweed may contribute to biological activities such as antioxidant, antimicrobial, and anti-inflammatory effects, all of which are important in the wound-healing process.

The present article aimed to analyze the main mechanisms involved in wound repair and to evaluate the potential of a new marine-derived biocomposite, prepared in two formulations, F1 and F2. Both formulations were based on collagen peptides extracted from jellyfish, chitosan obtained from marine stone crabs, and bioactive extracts from the green algae *C. vagabunda*. The in vitro studies performed on BALB/3T3 fibroblast cells and HaCaT keratinocytes showed encouraging results, supporting the potential of these formulations for tissue repair applications.

However, further studies are still needed before practical use can be considered. In vivo investigations are necessary to confirm the wound-healing effects under physiological conditions and to complete the biological profile of the proposed biocomposites. Additional research should also address remaining uncertainties related to the structure of the materials, the reproducibility and difficulty of bioactive compound extraction, and the cytotoxicity profile of the extracts, as these aspects may influence the development of future therapeutic solutions.

At the same time, more extensive strategic initiatives at the European level could support the sustainable harvesting and use of jellyfish and green algae, transforming these marine resources into valuable sources of natural bioactive compounds for biomedical applications. Overall, jellyfish, stone crabs, and green algae represent promising marine resources for the development of new wound-healing therapies, including collagen peptide-based biomaterials, polysaccharide-based treatments, topical pharmaceutical formulations, and tissue-engineering approaches.

## Figures and Tables

**Table 1:** Proximate composition of green algae *C. vagabunda* and collagen from jellyfish *R. pulmo* from the Black Sea.

Characteristics	*C. vagabunda*			Hydrogels Extract JPCs from *R. pulmo*	
	Algal Extract	References		With 10% Pepsin	Reference
		[53]	[54]		[38]
Ash 600–800 °C % (DW)	23.89 ± 0.94	24.63 ± 0.84	26.38 ± 0.31	0.85 ± 0.15	0.55 ± 0.1
Moisture % (DW)	9.12 ± 0.52	11.98 ± 0.84	5.71 ± 0.92	18.6 ± 0.18	15.1 ± 0.1
Carbohydrates % (DW)	62.22 ± 1.34	62.37 ± 1.74	58.45 ± 0.5	0.65 ± 1.28 W 0.28 ± 0.1 G	0.59 ± 1.25 W; 0.25 ± 0.65 G
Sulphates (%)	68.94 ± 1.25	68.65 ± 1.78	67.92 ± 0.53	-	-
Total nitrogen (%)	2.41 ± 0.31	2.39 ± 0.26	2.45 ± 0.02	-	-
Proteins % (DW)	15.07 ± 0.51	14.95 ± 0.92	15.43 ± 0.36	61.25 ± 1.25 W	60.48 ± 1.72 W
Collagen content % (DW)	-	-	-	57.5 ± 0.25	57.1 ± 0.6
Lipid % (DW)	3.15 ± 0.85	2.86 ± 0.75	3.85 ± 0.47	4.3 ± 0.55	4.9 ± 0.81 W;
Total dietary fiber % (DW)	62.45 ± 1.25	63.35 ±1.24	61.56 ± 1.5	-	-
Insoluble fiber %(DW)	36.54 ± 1.34	34.68 ± 0.82	38 ± 2.68	-	-
Soluble fiber % (DW)	25.91 ± 1.28	28.67 ± 1.75	22.92 ± 1.66	-	-

DW—dry weight; G—gonads; W—the whole.

**Table 2:** Physico-chemical parameters of chitosan.

Physico-Chemical Parameter	Chitosan CT	Reference [47]
Moisture %	8.42%	8.54%
Ash %	2.05%	2.23%
Degree of deacetylation (DD)	68.9%	71.5%
Molecular Mass (Mw)	9.05 × 10^5^ g/mol	8.98 × 10^5^ g/mol
pH value	6.8	6.9
Solubility %	70.3%	74.75%

**Table 3:** Composition of amino acids from jellyfish collagen.

Amino Acids	*R. pulmo* from Black Sea Coast Residues/1000 Residues		*R. pulmo* from Goa Coast India %
		References	
		[38]	[37]
Tissue (Whole body)			
Essential aminoacids (EAAs)			
Arginine (*Arg*)	5.8	6.2	5.63
Cystine (*Cys*)	1.8	1.2	-
Glutamic acid (*Glu*)	14.8	15.2	13.46
Glycine (*Gly*)	30.7	33.4	29.34
Histidine (*His*)	0.4	0.6	-
Leucine (*Leu*)	8.1	8.6	6.35
Lysine (*Lys*)	5.7	6.3	4.62
Proline (*Pro*)	3.2	3.9	2.97
Hydroxiproline (*Hyp*)	3.1	3.65	4.82
Threonine (*Thr*)	6.5	5.25	3.18
Triptophan (*Trp*)	2.9	2.8	4.72
Tyrosine (*Tyr*)	3.2	3.90	1.77
Valine (*Val*)	3.5	4.9	2.8
Non-essential aminoacids (NEAAs)			
Alanine (*Ala*)	8.2	6.9	10.38
Aspartic acid (*Asp*)	9.5	6.65	10.91
Serine (*Ser*)	0.9	1.7	-

We wrote the abbreviations for amino acids in italics, this being in accordance with the literature.

**Table 4:** The content of flavonoid and phenolic compounds from *C. vagabunda* and from jellyfish collagen from *R. pulmo*.

Sample	TPC (mg GAE/100 g d.w.)		TFC (mg CE/100 g d.w.)	
		References		Reference
*C. vagabunda*	405.5 ± 1.21	409.8 ± 1.68 [50]	15.6 ± 1.78	12.3 ± 1.78 [50]
JPCs from *R. pulmo*	1.54 ± 0.29	2.07 ± 0.31 [34]	-	-

**Table 5:** Individual polyphenolic compounds in *C. vagabunda* and *R. pulmo* extracts.

Type of Acid	Mean Value for Extract ALG ± SD mg/100 g f.w.	Percentage for Extract ALG %	Reference [55]	Mean Value for JPC ± SD mg/100 g f.w.	Percentage for JPCs %
Gallic Acid	8.2 ± 0.2	2.81	7.9 ± 0.1	5.84 ± 0.02	88.75
Protocatechuic Acid	72.1 ± 0.2	24.70	75.1 ± 0.03	-	-
Gensitic Acid	162.1 ± 0.3	55.55	155.3 ± 0.15	-	-
p-Hydroxy-benzoic Acid	5.2 ± 0.1	1.78	2.6 ± 0.01	-	-
Vanillic Acid	13.3 ± 0.05	4.55	12.8 ± 0.1	-	-
Caffeic Acid	12.4 ± 0.2	4.25	11.7 ± 0.09	-	-
Caftaric Acid	-	-	-	0.24 ± 0.01	3.65
Feluric Acid	4.1 ± 0.1	1.40	4.9 ± 0.2	-	-
Salicylic Acid	14.5 ± 0.2	4.96	13.1 ± 0.1	-	-
Syringic Acid	-	-	-	0.50 ± 0.009	7.60

**Table 6:** Organoleptic characteristics for new formulations intended for healing.

Appearance	Color	Appearance
Hidrogel from collagen peptides from jellyfish *R. pulmo*	White	Gelatin viscous
Hidrogel from collagen peptides from jellyfish *R. pulmo*, chitosan and from marine crabs, 1:1 (*v*/*v*)	White	Gelatin viscous
Formula F1 (JPC-CT-ALG), collagen peptide hydrogel from *R. pulmo* with marine chitosan hydrogel (1:1 *v*/*v*) and with hydroalcoholic extract of *C. vagabunda* 10%	Yellowish white	Gelatin
Formula F2 (JPC-CT-ALG), collagen peptide hydrogel from *R. pulmo* with marine chitosan hydrogel (1:1 *v*/*v*) and with hydroalcoholic extract of *C. vagabunda* 20%	Yellowish white	Gelatin viscous

**Table 7:** Minimal inhibitory concentration (MIC) of green algae extracts.

Bacterial Strains		MIC (µg/mL)		
	JPC from *R. pulmo*	CT from Marine Crabs	ALG *C. vagabunda*	F (JPC-CTALG)
*S. aureus*	25	25	25	25
*E. coli*	25	25	25	25
*K. pneumonia*	50	50	50	50
*S. epidermidis*	50	50	50	50
*P. mirabilis*	>100	>100	>100	>100

The results are the mean ± SD for *n* = 3.

**Table 8:** Equations used in rheological study.

Viscosity ɳ (cP) Depending on Shear Speed D (s^−1^)	Shear Speed D (s^−1^) in Correlation with the Selected Rotation Speed ω (rpm)	Shear Stress τ (Pa) Depending on Viscosity ɳ (cP) and Shear Speed D (s^−1^)	Shear Speed D (s^−1^) Depending on Shear Stress τ (Pa)
ɳ = f(D) (9)	D = ω * R (10)	τ = ɳ * D (11)	D = f(τ) (12)

## Data Availability

The original contributions presented in the study are included in the article; further inquiries can be directed to the corresponding authors.

## Abbreviations

The following abbreviations are used in this manuscript:

JPCs	Jellyfish Peptide Collagen from *Rhizostoma pulmo*
ALG	Algae (green algae *Cladophora vagabunda*)
CT	Chitosan from Pachygrapsus Mormoratus rock crabs
JPC-CT-ALG	Composite Jellyfish Peptides-Chitosan-Algae
DC	Circular Dichroism Spectrum
SDS-PAGE	Sodium Dodecyl Sulfate–Polyacrylamide Gel Electrophoresis
FT-IR	Fourier-Transform Infrared Spectroscopy
PSC	Pepsin Soluble Collagen
TPC	Total Phenolic Content
TFC	Total Flavonoid Content
DPPH	2,2-diphenyl-1-picrylhydrazyl
MIC	Minimal Inhibitory Concentration
BALB/3T3	A fibroblast cell line
HaCaT	Keratinocyte cell line
ROS	Reactive Oxygen Species
ECM	Extracellular Matrix
TGF-α	Transforming Growth Factor-Alfa
TGF-β	Transforming Growth Factor-Beta
FGF	Fibroblast Growth Factor
PDGF	Platelet-Derived Growth Factor
VEGF	Vascular Endothelial Growth Factor
